# Baseline trust moderates risk appraisal in guided aerial adventure sports: a three-wave field study

**DOI:** 10.3389/fpsyg.2026.1868476

**Published:** 2026-08-25

**Authors:** Xiangyu Wang, Yao Pan, Xiaoyun Wang

**Affiliations:** 1 School of Physical Education, Sichuan Normal University, Chengdu, China; 2 Key Laboratory of Sports and Health Tourism Technology Research and Development, Department of Culture and Tourism of Sichuan Province, Chengdu, China; 3 School of Physical Education, Southwest Petroleum University, Chengdu, China

**Keywords:** aerial adventure sports, challenge appraisal, flow, perceived safety, re-participation intention, risk perception, threat appraisal, trust

## Abstract

Guided aerial adventure sports combine environmental uncertainty with reliance on pilots, equipment, and safety systems, making trust a plausible resource in risk appraisal. This three-wave field study examined whether baseline trust moderated associations between pre-activity risk perception and post-activity threat and challenge appraisals, and how these appraisals related to perceived safety, flow, and later re-participation intention. Participants at commercial sites in the Western Sichuan Plateau, China completed surveys before the activity (T1), 15–30 min after landing (T2), and approximately 21 days later (T3; linked *n* = 424). An eight-factor confirmatory model fit the data well, chi-square(1,297) = 1,523.42, CFI = 0.989, TLI = 0.988, RMSEA = 0.020, 90% CI [0.016, 0.024], and SRMR = 0.027. In standardized observed-score models, T1 risk perception was positively associated with T2 threat appraisal (beta = 0.443) and challenge appraisal (beta = 0.555). Baseline trust weakened the risk perception-threat association (interaction beta = −0.307) and strengthened the risk perception-challenge association (interaction beta = 0.250). Threat appraisal was negatively associated with perceived safety and flow, whereas challenge appraisal was positively associated with both. Perceived safety (beta = 0.432) and flow (beta = 0.442) were positively associated with T3 re-participation intention. The total conditional indirect association shifted from negative at low trust (estimate = −0.186, 95% CI [−0.256, −0.114]) to positive at high trust (estimate = 0.308, 95% CI [0.235, 0.384]). The pattern remained stable across HC3, inverse-probability-weighted, leave-one-site/activity, and influential-case analyses. These findings position baseline trust as an appraisal resource that helps organize perceived risk as threat or challenge, with implications for psychologically informed risk communication in guided aerial sport.

## Introduction

Guided aerial adventure sports such as tandem and powered paragliding combine bodily arousal, environmental uncertainty, and natural settings. Adventure-based and ecological perspectives frame these activities as psychologically meaningful encounters rather than scenic consumption alone (Boudreau et al., 2022; Brymer et al., 2024; Janowski et al., 2025; Turecek et al., 2025). Evidence from aerial sports identifies launch, flight exposure, landing, perceived risk, anxiety, and self-confidence as central to participation experience (Wilkes et al., 2022; McNeil et al., 2024). Guided formats add a distinctive dependency: participants confront altitude, wind, equipment, and pilot coordination while retaining limited technical control. This combination makes guided aerial adventure a useful setting for examining how risk appraisal shapes optimal experience and future participation.

Perceived risk is a psychological judgment as well as an attribute of the activity. Travel, tourism, and sport-tourism research links perceived risk with travel intention, revisit intention, destination involvement, adventure decisions, and mountaineering re-participation (Liang and Xue, 2021; Jiang et al., 2022; Johann et al., 2022; Li et al., 2023; Nam and Yang, 2025; Teng et al., 2025; Gu et al., 2026). Framing, psychological distance, and individual differences further influence how uncertainty is interpreted (Buelow et al., 2022; Xie et al., 2023; Zhang et al., 2023). In adventure contexts, however, risk may signal potential harm or contribute to excitement and mastery when demands appear manageable. The central question is therefore how participants appraise perceived risk as the activity unfolds.

Challenge-threat theory offers a mechanism for this divergence. Contemporary appraisal approaches propose that motivated experience depends on how individuals evaluate situational demands, available resources, and control (Meijen et al., 2020; Tomaka and Magoc, 2021; Lin et al., 2026). A challenge appraisal is more likely when demands appear meaningful and manageable; a threat appraisal is more likely when demands appear to exceed available resources. Sport and stress research links these appraisals with wellbeing, performance evaluation, personality, anxiety, and coping flexibility (Mansell, 2021; Turner et al., 2021; Chen and Qu, 2023; Jewiss et al., 2023; Karademir et al., 2026). Measurement evidence also supports treating challenge and threat as related but distinct dimensions, not as opposite ends of a single continuum (Rossato et al., 2018).

Trust is especially salient in guided aerial adventure because participants depend on pilots, operators, equipment preparation, weather judgment, and safety procedures. Tourism research identifies trust as a condition of risk interpretation and travel-related decision making under uncertainty (Sarfraz et al., 2022; Ameen et al., 2024). In appraisal terms, baseline trust may serve as an externally anchored coping resource: confidence in the social and technical system can make demanding risk cues appear more manageable. Low baseline trust should align perceived risk more strongly with threat, whereas high baseline trust should favor a challenge appraisal. Post-activity trust is treated separately because it reflects the completed experience rather than the resource state present before participation.

Perceived safety and flow are proximal experience states that may connect appraisal with later intention. Perceived safety denotes subjective confidence that the activity was manageable, professionally supported, and governed by credible procedures; it does not denote objective risk elimination. Safety research treats this judgment as staged, socially constructed, and behaviorally relevant (Zou and Yu, 2022; Xie et al., 2025), while risk-message studies link safety interpretation with intention under uncertain travel conditions (Xie et al., 2023; Teng et al., 2025). Flow denotes deep absorption, focused attention, perceived control, and intrinsic enjoyment. Across sport, adventure recreation, and tourism, flow is associated with continued participation, behavioral intention, and psychological recovery (Lu et al., 2022; Swann et al., 2022; Jackson et al., 2023; Bayram et al., 2025; Song et al., 2025).

Re-participation intention is a consequential behavioral intention in recreational sport and tourism. Prior studies connect it with flow, destination involvement, perceived risk, challenge perception, and motivation (Johann et al., 2022; Bayram et al., 2025; Lin et al., 2026), and moderated mediation has been used to model risk and re-participation in mountaineering (Gu et al., 2026). Three gaps remain. First, guided aerial sport places unusual weight on interpersonal and technical trust. Second, single-wave designs conflate anticipatory risk, post-activity appraisal, and later intention. Third, models that treat risk as uniformly negative overlook the possibility that perceived resources redirect risk toward challenge.

**Figure 1:**
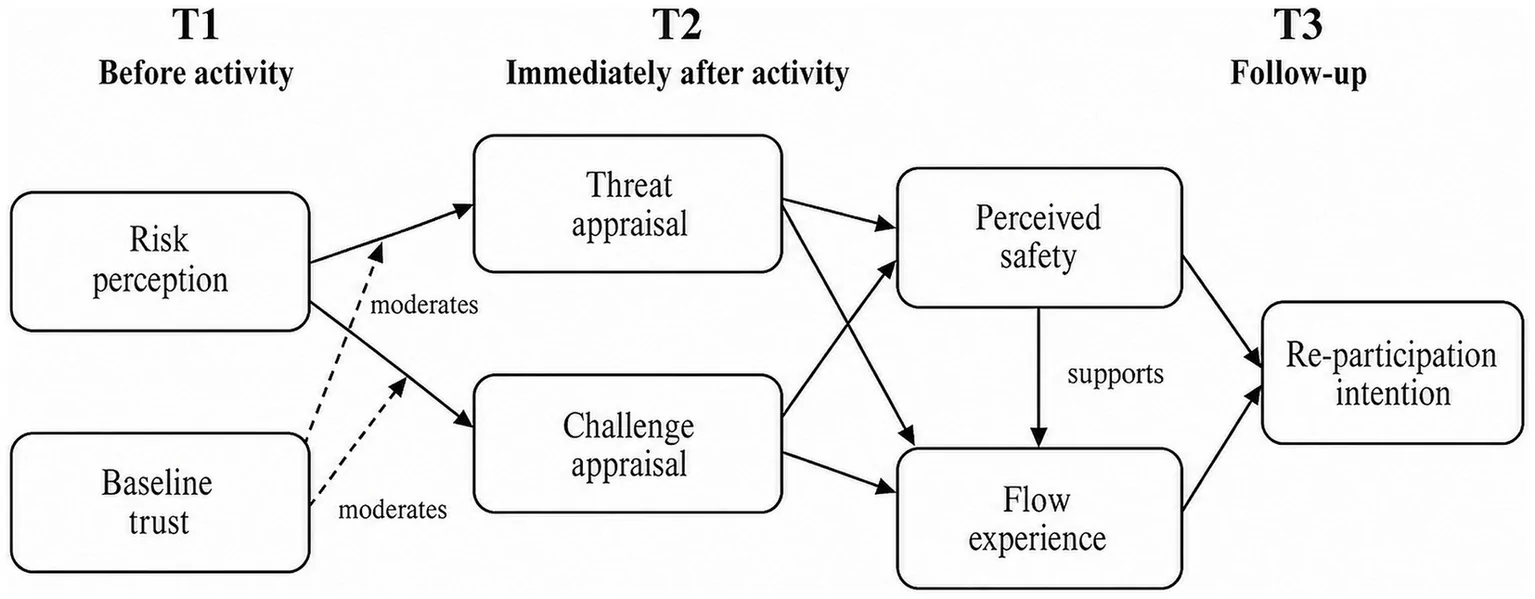
Conceptual model of the three-wave risk appraisal pathway. The figure displays the hypothesized ordering from T1 pre-activity risk perception and baseline trust to T2 post-activity threat appraisal, challenge appraisal, perceived safety, and flow, and then to T3 re-participation intention. Dashed paths indicate moderation by T1 baseline trust. Solid paths indicate theory-specified associations tested in observed-score path models; cross-wave paths are temporally ordered, whereas the perceived safety to flow path is a contemporaneous T2 association. T1 = before activity; T2 = 15–30 min after landing; T3 = approximately 21 days after activity. Covariates included age, gender, education, income, prior aerial adventure experience, broader outdoor adventure experience, sensation seeking, activity type, and site; covariates, item indicators, residuals, and robustness-only paths are omitted from the display.

The present study addressed these gaps with a three-wave field survey of guided aerial adventure sport participants in the Western Sichuan Plateau. T1 assessed pre-activity risk perception and baseline trust, T2 assessed post-activity appraisals and experience states, and T3 assessed re-participation intention. The model links T1 risk perception with T3 intention through T2 threat appraisal, challenge appraisal, perceived safety, and flow, with T1 baseline trust moderating the initial appraisal paths. Because no focal variable was experimentally manipulated, the hypotheses concern temporally ordered associations. Figure 1 presents the conceptual model.

Risk perception is expected to be psychologically ambivalent. It signals demands arising from altitude, wind, launch and landing uncertainty, equipment dependence, and bodily exposure (Meijen et al., 2020; Lin et al., 2026). Evidence from travel, sport tourism, and adventure tourism likewise links perceived risk with both avoidance-oriented and participation-oriented decisions when framing, psychological distance, desired risk, and individual differences are considered (Liang and Xue, 2021; Buelow et al., 2022; Johann et al., 2022; Xie et al., 2023; Zhang et al., 2023; Li et al., 2023; Nam and Yang, 2025; Teng et al., 2025; Gu et al., 2026). Risk should therefore align with threat when demands appear to exceed coping resources, but with challenge when those demands appear meaningful and manageable.

H1a: Higher T1 risk perception will be positively associated with T2 threat appraisal.

H1b: Higher T1 risk perception will be positively associated with T2 challenge appraisal.

Baseline trust is expected to operate as a secondary-appraisal resource. In guided aerial adventure, participants rely on pilots, operators, equipment checks, weather judgments, and emergency procedures. Safety-related trust may therefore compensate for limited technical control and increase the perceived availability of coping resources (Sarfraz et al., 2022; Ameen et al., 2024). Baseline trust should consequently condition whether perceived risk is interpreted as unmanaged danger or as a demanding but supported activity.

H2a: T1 baseline trust will moderate the association between T1 risk perception and T2 threat appraisal, such that the positive association will be weaker at higher levels of baseline trust.

H2b: T1 baseline trust will moderate the association between T1 risk perception and T2 challenge appraisal, such that the positive association will be stronger at higher levels of baseline trust.

Threat and challenge appraisals should have divergent associations with proximal experience states. Sport psychology and stress research links challenge-threat processes with performance evaluations, wellbeing, anxiety, coping flexibility, and appraisal-related individual differences (Mansell, 2021; Tomaka and Magoc, 2021; Turner et al., 2021; Chen and Qu, 2023; Jewiss et al., 2023; Karademir et al., 2026). Threat appraisal is expected to involve defensive monitoring, worry, and attention to possible harm, which should correspond with lower perceived safety and weaker flow. Challenge appraisal should instead involve perceived manageability, approach motivation, and task engagement, supporting perceived safety and the challenge-skill balance central to flow.

H3a: T2 threat appraisal will be negatively associated with T2 perceived safety and T2 flow.

H3b: T2 challenge appraisal will be positively associated with T2 perceived safety and T2 flow.

Perceived safety is expected to be positively associated with flow. Confidence that an activity is professionally managed can reduce defensive monitoring and free attention for the experience itself. Because flow is intrinsically rewarding and predicts continued engagement across sport and adventure settings (Lu et al., 2022; Swann et al., 2022; Jackson et al., 2023; Bayram et al., 2025; Song et al., 2025), both perceived safety and flow should be positively associated with later re-participation intention.

H4a: T2 perceived safety will be positively associated with T2 flow.

H4b: T2 perceived safety and T2 flow will each be positively associated with T3 re-participation intention.

The integrated model proposes that pre-activity risk perception becomes associated with later intention through post-activity appraisal and experience. This account connects risk-intention research in tourism and outdoor sport (Johann et al., 2022; Nam and Yang, 2025; Teng et al., 2025; Gu et al., 2026) with flow-based explanations of repeated recreational participation (Jackson et al., 2023; Bayram et al., 2025).

H5: The indirect association between T1 risk perception and T3 re-participation intention through T2 threat appraisal, challenge appraisal, perceived safety, and flow will be conditional on T1 baseline trust, with the total indirect association becoming more positive as baseline trust increases.

## Materials and methods

### Study design

A three-wave prospective field survey assessed participants before the aerial adventure activity (T1), 15–30 min after landing (T2), and approximately 21 days later (T3; response window: 14–28 days). The study was observational and naturalistic. Commercial operators and site managers retained responsibility for participant assignment, safety procedures, and flight decisions; the research team did not alter operations.

### Ethics approval and consent

This study was conducted in accordance with the ethical principles of the Declaration of Helsinki. Under Article 32(2) of the Measures for Ethical Review of Life Sciences and Medical Research Involving Humans issued by the National Health Commission of the People’s Republic of China, the Ministry of Education, the Ministry of Science and Technology, and the National Administration of Traditional Chinese Medicine, research using anonymized information data may be exempt from formal ethical review when it does not cause harm to human participants and does not involve sensitive personal information or commercial interests. The School of Physical Education, Sichuan Normal University assessed the project under applicable institutional requirements for minimal-risk anonymous questionnaire research and confirmed that formal review by an institutional ethics committee was not required. The present study met the exemption conditions because it used an anonymous, voluntary three-wave field survey with adult guided aerial adventure sport participants, involved no clinical intervention, deception, biological sampling, clinical treatment, health-record access, minors, identifiable questionnaire dataset, sensitive personal information, commercial sponsor, or transfer of identifiable data to any operator or commercial entity, and all questionnaire data were de-identified before analysis. Participants created or received anonymous linking codes for wave matching. Contact information voluntarily provided for T3 follow-up was stored separately from questionnaire responses, password-protected, accessible only to the research team, and not included in the research dataset. Before completing the survey, participants were informed of the study purpose, the voluntary nature of participation, their right not to provide follow-up contact information, the intended use of the data, the anonymity and de-identification of questionnaire responses, and the separate handling of follow-up contact information. Submission of the completed questionnaire was taken as informed consent. The relevant regulatory document is available at: https://www.nhc.gov.cn/wjw/c100375/202302/902b4a1dc3af4aba862a6387e6e376dc.shtml.

### Setting

Recruitment took place at commercial guided aerial adventure sport sites in the Western Sichuan Plateau, China, including operations in the Kangding, Litang, and Xiaojin/Siguniangshan areas. The core activity frame comprised tandem paragliding, powered paragliding, and other commercial activities that involved a discrete guided aerial episode, dependence on a pilot or operator and equipment system, and feasible T1 and T2 data collection. Passive sightseeing-only activities, including hot-air-balloon and light-aircraft sightseeing records in the broader field database, were excluded. Additional sites were retained only when they met the same operational, fieldwork, and ethics criteria.

The Western Sichuan Plateau provided repeated access to guided aerial activities conducted in high-altitude, open-terrain settings where environmental uncertainty and operator dependence were salient. Recruitment occurred only during operator-approved activity periods, and operators retained all weather-related go/no-go decisions. The research team did not collect instrument-level weather records or intervene in operational safety procedures.

### Participants and recruitment

Eligible recreational aerial adventure sport participants were recruited on site using consecutive convenience sampling during eligible operating days. Inclusion criteria were: (1) age 18 years or older; (2) participation in a guided tandem paragliding, powered paragliding, or comparable guided aerial adventure activity at the study site; (3) ability to read and complete a Chinese questionnaire; (4) completion of T1 before the activity and T2 after landing; and (5) willingness to provide a contact method for T3 follow-up. Exclusion criteria were: (1) professional pilots, coaches, site staff, or research staff; (2) participation in non-comparable passive sightseeing activities only; (3) inability to provide informed consent; (4) duplicate responses; (5) incomplete linking information across waves; and (6) response patterns indicating careless or invalid responses according to predefined data-quality rules.

Recruitment aimed to yield at least 400 linked T3 cases, allowing estimation of adjusted paths, interaction terms, and bootstrap confidence intervals after anticipated attrition. The final core sample contained 424 linked T1-T2-T3 cases. With 16 predictors in the most parameterized focal regression, this sample provided 80% power at alpha = 0.05 to detect a one-degree-of-freedom effect of approximately *f*^2^ = 0.019 (partial *r* = 0.135). This sensitivity calculation characterizes the minimum detectable effect; it does not validate the observed effects retrospectively.

### Procedure

Before data collection, the research team contacted site managers and operators to confirm activity type, recruitment feasibility, safety boundaries, and non-interference with operations. Field investigators were trained to approach participants only during appropriate waiting periods and to avoid interrupting safety briefings, equipment checks, weather decisions, or pilot instructions.

At T1, participants were approached after activity registration but before the flight or immediately before formal activity preparation, depending on site workflow. After reading the participant information sheet and providing consent, participants completed measures of risk perception, baseline trust, sensation seeking, prior aerial adventure experience, previous plateau exposure, and demographics. Participants created or received a unique anonymous linking code. Gender was self-reported using inclusive response options and was used only as an adjustment variable.

The research team retained the original recruitment logs, participant information materials, de-identification procedure, data-cleaning record, and analysis scripts in a protected project archive.

At T2, participants completed the second survey 15–30 min after landing, after immediate operational procedures had ended and when they were physically comfortable. Measures assessed threat appraisal, challenge appraisal, perceived safety, post-activity trust, and flow. Site code and activity type were available for every analyzed case. Technical weather parameters and exact flight duration were not consistently available; therefore, they were not reported as distributions or modeled. Site and activity type were included as covariates and examined in leave-one-site and leave-one-activity analyses. Investigators did not request immediate survey completion from participants reporting distress, physical discomfort, or an urgent need to leave.

At T3, participants received an online follow-up survey approximately 21 days after the activity, with reminders sent within the 14-28-day response window. T3 measures assessed re-participation intention, recommendation intention, and any subsequent relevant experience. Contact information was stored separately from survey responses and was used only for follow-up delivery unless the participant provided separate permission for future contact.

### Measures

All substantive items used five-point Likert-type response options ranging from 1 (strongly disagree) to 5 (strongly agree), with higher scores indicating higher levels of the construct. Scale scores were calculated as item means when valid item responses were available. Established scales and published measurement frameworks were used where appropriate. Constructs requiring aerial-sport wording were assessed with context-adapted items designed to preserve the target construct while matching guided aerial adventure participation. Supplementary Table S1 provides English reporting wording and variable mapping for the project items; original sources, adaptation decisions, and the complete Chinese questionnaire wording were retained in the study records as measurement documentation.

### Risk perception at T1

Pre-activity risk perception was assessed with seven items capturing the extent to which participants perceived the upcoming aerial adventure activity as involving environmental risk, bodily or physiological risk, equipment-related risk, and operator or management risk. The item domains covered weather and wind uncertainty, launch and landing exposure, bodily discomfort or fear responses, equipment reliability, and operator management. Items were adapted from sport-tourism and tourism-intention risk perception research (Jiang et al., 2022; Johann et al., 2022; Nam and Yang, 2025), sport and adventure tourism decision research (Li et al., 2023; Teng et al., 2025; Gu et al., 2026), and air-sport evidence on paragliding exposure and perceived risk (Wilkes et al., 2022). The construct captured subjective judgment rather than objective safety level.

### Baseline trust at T1

Baseline trust was assessed with 10 items measuring pre-activity confidence in the operator, pilot, equipment preparation, and safety management. Item content reflected perceived pilot competence, care for participant safety, communication clarity, procedural reliability, equipment preparation, emergency readiness, and overall trust. The trust construct was informed by tourism studies linking trust, perceived risk, safety practices, and travel-related behavioral outcomes (Sarfraz et al., 2022; Ameen et al., 2024).

### Sensation seeking at T1

Sensation seeking was assessed with eight brief items capturing preferences for novelty, intensity, stimulation, and unusual experiences. It was included as a covariate because risk-related dispositions may influence both adventure participation and appraisal.

### Challenge and threat appraisals at T2

Post-activity challenge and threat appraisals were assessed with six items each. Challenge-appraisal items captured manageable demand, achievement value, approach motivation, and perceived capability, whereas threat-appraisal items captured anticipated harm, loss of control, worry, and perceived resource insufficiency. The two dimensions were treated as related but distinct constructs rather than opposite ends of one continuum. Item content was informed by the Challenge and Threat in Sport Scale and by recent sport psychology and stress studies applying challenge and threat appraisal to wellbeing, performance, anxiety, coping, and individual-difference outcomes (Rossato et al., 2018; Mansell, 2021; Tomaka and Magoc, 2021; Turner et al., 2021; Chen and Qu, 2023; Jewiss et al., 2023; Karademir et al., 2026). Because the original CAT-Sport was developed for competitive sport anticipation, final items were context-adapted for recreational aerial adventure and evaluated through confirmatory factor analysis before structural testing.

### Perceived safety at T2

Perceived safety was assessed with six items measuring the participant’s subjective sense that the flight was manageable, professionally supported, procedurally clear, and protected by credible safety arrangements. Item wording was informed by destination-safety and tourist-metasafety research (Zou and Yu, 2022; Xie et al., 2025) and by risk-message work that treats perceived safety as part of risk-intention processing (Xie et al., 2023). Items focused on subjective experience and did not ask participants to evaluate technical compliance or objective risk. Higher scores indicated stronger perceived safety during the activity.

### Flow at T2

Flow during the activity was assessed with eight event-specific items informed by recent sport, exercise, tourism, and adventure recreation flow research (Lu et al., 2022; Swann et al., 2022; Jackson et al., 2023; Bayram et al., 2025; Song et al., 2025). Item content covered dimensions such as concentration, sense of control, challenge-skill balance, merging of action and awareness, time transformation, and autotelic experience.

### Post-activity trust at T2

Post-activity trust was assessed with six items measuring the participant’s trust in the pilot, operator, equipment preparation, and safety procedures after the completed experience. Because post-activity trust was measured after the activity, it was not used as the primary moderator of the T1 risk perception to T2 appraisal paths. It was included in robustness analyses to examine whether the main pattern remained after accounting for trust formed or updated during the activity.

### Re-participation intention and recommendation intention at T3

Re-participation intention was assessed with four items measuring willingness to participate in the same or a similar aerial adventure sport in the future. Recommendation intention was assessed with three items measuring willingness to recommend the activity to others. Re-participation items were adapted from sport-tourism and recreation studies linking flow, involvement, perceived risk, challenge perception, and behavioral intention (Jiang et al., 2022; Johann et al., 2022; Bayram et al., 2025; Gu et al., 2026; Lin et al., 2026). Recommendation-intention wording was informed by behavioral-intention research in tourism and sport settings (Lu et al., 2022; Nam and Yang, 2025). Re-participation intention was the primary T3 outcome; recommendation intention was treated as a secondary robustness outcome.

### Covariates

Covariates were age, self-reported gender, education, income category, prior aerial adventure experience, broader outdoor adventure experience, sensation seeking, activity type, and site. Technical weather parameters and exact flight duration were not consistently available; therefore, they were not reported as distributions or modeled. Previous high-altitude exposure, self-rated health, waiting time, and discomfort were also unavailable for some participants and were treated as unmeasured field characteristics.

### Data quality and missing data

Data were screened before analysis according to predefined rules. Invalid responses included failed age, eligibility, or consent screens; duplicate submissions or duplicate anonymous linking codes; failure to link waves with a valid anonymous code; impossible age values; completion times too short for attentive responding; invariant responses across long item blocks; failed attention checks; and responses with excessive missing data in focal variables. Exclusion rules were applied before hypothesis testing and documented in the participant flow results.

Attrition was examined at both follow-up transitions. First, the 424 retained T3 participants were compared with all 105 non-retained participants in the core T1 frame on baseline demographic and psychological variables. Second, among the 503 participants who completed T2, the 424 retained participants were compared with the 79 participants lost before T3 on threat appraisal, challenge appraisal, perceived safety, flow, and post-activity trust. The core analysis was restricted to tandem paragliding, powered paragliding, and comparable guided aerial adventure activities. The broader sensitivity frame retained all 600 T3-matched field records, including non-core sightseeing-oriented projects, but these records were not treated as part of the core guided aerial adventure sport population.

Because retention differed on focal T1 variables, the primary inverse-probability weighting sensitivity analysis modeled T3 retention from T1 demographic, experience, risk perception, baseline trust, sensation seeking, site, and activity-type variables. A second post-T2 retention analysis, restricted to the 503 T2 completers, added T2 threat appraisal, challenge appraisal, perceived safety, flow, and post-activity trust to the same baseline predictor set. The T1-only and T1 + T2 retention models were compared on the same T2-completer frame using a likelihood-ratio test and area under the receiver operating characteristic curve. Stabilized weights from each specification were trimmed at the 1st and 99th percentiles before weighted estimation of the final T3 model. These analyses address selection on measured baseline and post-activity states; they cannot adjust for unrecorded physical discomfort, adverse operational events, later interpersonal influences, or other unmeasured reasons for nonresponse.

### Statistical analysis

Analyses were conducted in Python 3.13.3 using pandas 2.3.3, NumPy 2.3.5, SciPy 1.17.0, statsmodels 0.14.6, and semopy 2.3.11. Tests were two-sided with alpha = 0.05 and 95% confidence intervals, and interpretation emphasized effect sizes alongside *p* values. Theory-specified paths, moderation terms, and conditional indirect associations were primary analyses. Recommendation intention, post-activity trust adjustment, the broader field sample, leave-one-site/activity analyses, robust standard errors, and influential-case diagnostics were secondary or sensitivity analyses. Because inference centered on one theory-specified path model, no omnibus multiplicity correction was applied; secondary findings were interpreted descriptively.

Descriptive statistics, reliability estimates, and bivariate correlations were calculated for the focal variables. Internal consistency was evaluated with Cronbach’s alpha and McDonald’s omega for scales with at least three items. Attrition comparisons used standardized mean differences for continuous variables and Cramer’s V for gender. Among T2 completers, retained and non-retained participants were compared with Welch tests and pooled Cohen’s *d* values as selection diagnostics.

Confirmatory factor analysis evaluated risk perception, baseline trust, threat appraisal, challenge appraisal, perceived safety, flow, post-activity trust, and re-participation intention. Fit was assessed with chi-square, CFI, TLI, RMSEA with a 90% confidence interval, and SRMR. Standardized loadings, composite reliability, average variance extracted, and heterotrait-monotrait ratios assessed construct quality. Two alternative models merged perceived safety with flow and then perceived safety, flow, and intention to test whether the downstream constructs were reducible to a common factor. Common-method diagnostics comprised an unrotated first-factor test and a one-factor CFA. Supplementary parcel-level multi-group CFA screened configural, metric, and scalar invariance across activity types and sites; the screening was interpreted cautiously because the smallest site subgroup limited item-level estimation.

Structural paths were estimated from standardized scale scores. An item-level latent interaction with serial conditional indirect paths would have required substantially more parameters than the retained sample supported; the observed-score model therefore provided a transparent and reproducible specification for product terms, bootstrapping, inverse-probability weighting, and leave-one-site/activity analyses. CFA was reported separately as measurement evidence, and the structural coefficients remain uncorrected for measurement error.

The model included paths from T1 risk perception to T2 threat and challenge appraisals; from both appraisals to T2 perceived safety and flow; from T2 perceived safety to T2 flow; and from perceived safety and flow to T3 re-participation intention. T1 baseline trust moderated the risk perception-appraisal paths. All models adjusted for age, gender, education, income, prior aerial adventure experience, broader outdoor adventure experience, sensation seeking, activity type, and site. Because perceived safety and flow were measured at T2, their association was modeled as contemporaneous. The ordering reflects the theoretical view that felt safety supports absorption, but the data also permit reverse ordering, reciprocal reinforcement, or a shared positive-experience process. Alternative T3 models and a Wald contrast evaluated this overlap (Supplementary Table S5).

Indirect associations were estimated with 5,000 bootstrap resamples and evaluated at low (−1 SD), mean, and high (+1 SD) baseline trust. Adjusted marginal means fixed risk perception at its lower and upper quartiles and trust at its lower quartile, median, and upper quartile while averaging over the observed covariate distribution. Adjusted contrasts were standardized by the observed mediator standard deviation.

Diagnostic analyses added post-activity trust, substituted recommendation intention, applied T1-based and T1 + T2 inverse-probability weights, omitted each site or activity type in turn, and analyzed the broader field sample. Activity-specific means and tandem-versus-powered differences were descriptive. Breusch-Pagan tests assessed heteroskedasticity, HC3 standard errors were estimated for focal paths, and variance inflation factors and site-level intraclass correlations were inspected. Influential-case analysis excluded observations above Cook’s *D* = 4/*n* as a diagnostic specification.

## Results

### Participant flow and sample characteristics

**Figure 2:**
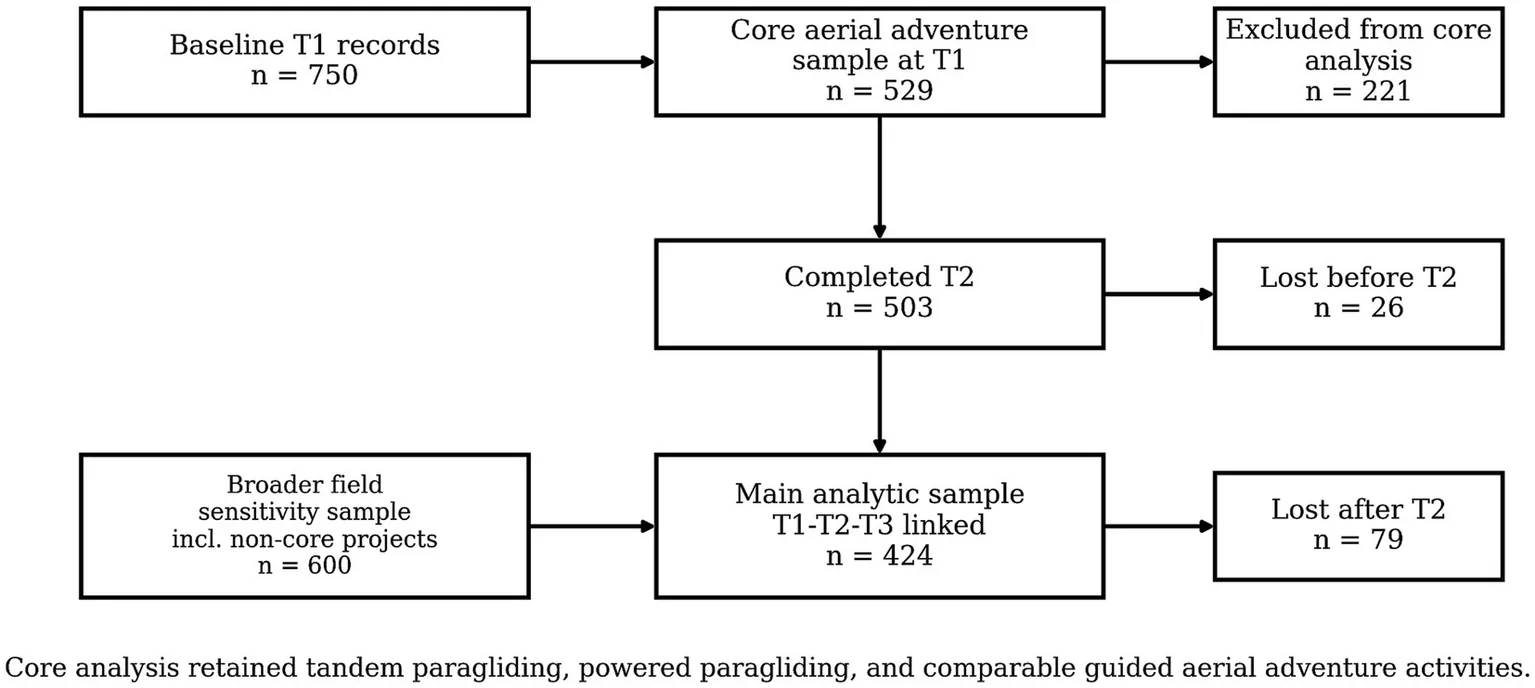
Participant flow across the three-wave field survey. The initial field record contained 750 T1 screening records. After eligibility and quality screening, 529 participants entered the core T1 baseline frame. Of these, 503 completed the T2 post-activity survey and 424 completed the T3 follow-up, yielding 424 retained cases for the primary three-wave analysis. Attrition included 26 participants lost before T2 and 79 participants lost after T2. The broader field sensitivity frame contained 600 T3-matched participants, including non-core low-altitude or sightseeing-oriented projects, and was used only as an auxiliary sensitivity-analysis frame rather than as an additional sequential exclusion step.

Of 750 T1 records, 529 met the core activity and data-quality criteria, 503 completed T2, and 424 completed T3 within the planned follow-up window. Twenty-six core participants were lost before T2 and 79 completed T2 but were not matched at T3. The final core analytic sample therefore comprised 424 participants with linked data across all three waves. Figure 2 presents participant flow and the broader field sensitivity sample, which included non-core low-altitude or sightseeing-oriented projects.

Participants had a mean age of 30.61 years (SD = 7.08; range = 18–55); 205 participants identified as female (48.3%), 210 as male (49.5%), and 9 as another category or preferred not to answer (2.1%). In the core analytic sample, 202 participants completed tandem paragliding, 133 completed powered paragliding, and 89 completed another comparable guided aerial adventure activity meeting the operational definition described above. The sample was recruited from five anonymized sites: S01 (*n* = 112), S02 (*n* = 96), S03 (*n* = 61), S04 (*n* = 124), and S05 (*n* = 31). A total of 211 participants (49.8%) reported no prior low-altitude or aerial adventure sport experience. Table 1 presents demographic and activity characteristics.

**Table 1:** Core sample flow and characteristics.

Indicator	*n* or statistic
Core T1 records	529
Core T2 completed	503
Core T3 matched/main analytic sample	424
Lost before T2	26
Completed T2 but not T3	79
Age, M (SD), range	30.61 (7.08), 18–55
Female	205 (48.3%)
Male	210 (49.5%)
Other/prefer not to answer	9 (2.1%)
First-time aerial adventure participants	211 (49.8%)
Tandem paragliding	202
Powered paragliding	133
Other comparable aerial adventure sport	89
Site S01/S02/S03/S04/S05	112/96/61/124/31

Attrition analyses compared the 424 T3 participants with 105 non-retained participants in the core T1 frame. The groups did not differ by age, *t* = 1.24, *p* = 0.216, *d* = 0.139; sensation seeking, *t* = −0.98, *p* = 0.331, *d* = −0.107; or gender distribution, chi-square(2) = 0.81, *p* = 0.668, Cramer’s V = 0.039. Retained participants nevertheless reported lower T1 risk perception (M = 3.13 vs. 3.80), *t* = −5.96, *p* < 0.001, *d* = −0.630, and higher T1 baseline trust (M = 4.02 vs. 2.84), *t* = 11.63, *p* < 0.001, *d* = 1.361. Retention was therefore selective with respect to both focal baseline variables.

Among the 503 T2 completers, 79 did not complete T3. Compared with those lost after T2, retained participants reported lower threat appraisal (M = 2.57 vs. 3.43, *d* = −0.848), higher challenge appraisal (M = 3.88 vs. 3.41, *d* = 0.505), higher perceived safety (M = 4.05 vs. 3.42, *d* = 0.718), higher flow (M = 3.87 vs. 3.11, *d* = 0.815), and higher post-activity trust (M = 4.07 vs. 3.29, *d* = 0.912); all Welch *p* values were <0.001. After adjustment for T1 predictors, adding these five T2 states did not improve retention-model fit, likelihood-ratio chi-square(5) = 4.03, *p* = 0.546; AUC changed from 0.873 to 0.874 (Supplementary Table S7).

### Descriptive statistics, reliability, and correlations

Table 2 presents means, standard deviations, reliability estimates, and key correlations. Cronbach’s alpha ranged from 0.897 to 0.963, and McDonald’s omega ranged from 0.898 to 0.963, indicating high internal consistency across the focal constructs.

**Table 2:** Descriptive statistics, reliability, and correlations of focal constructs.

(A) Descriptive statistics and reliability						
Construct	Items	*n*	M	SD	alpha	omega
T1 risk perception	7	424	3.13	1.07	0.957	0.957
T1 baseline trust	10	424	4.02	0.85	0.958	0.959
T1 sensation seeking	8	424	3.32	1.04	0.963	0.963
T2 threat appraisal	6	424	2.57	1.00	0.928	0.928
T2 challenge appraisal	6	424	3.88	0.92	0.936	0.937
T2 perceived safety	6	424	4.05	0.85	0.948	0.948
T2 flow	8	424	3.87	0.88	0.940	0.940
T2 post-activity trust	6	424	4.07	0.81	0.922	0.923
T3 re-participation intention	4	424	3.79	0.95	0.897	0.898
T3 recommendation intention	3	424	3.96	0.94	0.911	0.912

(B) Pearson correlations among focal constructs.								
Variable	1	2	3	4	5	6	7	8
1. RP	–							
2. BT	−0.247	–						
3. SS	0.281	0.025	–					
4. TH	0.570	−0.539	0.230	–				
5. CH	0.422	0.392	0.129	−0.074	–			
6. PS	−0.099	0.554	−0.035	−0.600	0.555	–		
7. FL	−0.093	0.504	−0.094	−0.497	0.589	0.629	–	
8. RI	−0.063	0.532	−0.035	−0.557	0.539	0.739	0.741	–

RP = risk perception; BT = baseline trust; SS = sensation seeking; TH = threat appraisal; CH = challenge appraisal; PS = perceived safety; FL = flow; RI = re-participation intention. Correlations are based on the core linked sample, *n* = 424.

Mean scores were 3.13 (SD = 1.07) for T1 risk perception, 4.02 (SD = 0.85) for baseline trust, 2.57 (SD = 1.00) for T2 threat appraisal, 3.88 (SD = 0.92) for challenge appraisal, 4.05 (SD = 0.85) for perceived safety, 3.87 (SD = 0.88) for flow, 4.07 (SD = 0.81) for post-activity trust, 3.79 (SD = 0.95) for T3 re-participation intention, and 3.96 (SD = 0.94) for recommendation intention. Risk perception correlated positively with threat appraisal (*r* = 0.570) and challenge appraisal (*r* = 0.422), but weakly with re-participation intention (*r* = −0.063). Perceived safety and flow were strongly correlated (*r* = 0.629), and re-participation intention correlated strongly with perceived safety (*r* = 0.739), flow (*r* = 0.741), and recommendation intention (*r* = 0.748). These correlations motivated the simultaneous and construct-overlap analyses.

### Measurement model

The hypothesized eight-factor measurement model showed good fit: chi-square(1,297) = 1523.42, *p* < 0.001, CFI = 0.989, TLI = 0.988, RMSEA = 0.020, 90% CI [0.016, 0.024], SRMR = 0.027. Standardized factor loadings ranged from 0.757 to 0.919. Composite reliability ranged from 0.897 to 0.959, and average variance extracted ranged from 0.663 to 0.759. HTMT values were below 0.85 for all construct pairs; the largest value was 0.806 for flow and re-participation intention, and the HTMT value for threat appraisal and challenge appraisal was 0.081. Challenge appraisal and threat appraisal were empirically distinguishable, with a small and non-significant latent correlation (*r* = −0.083, *p* = 0.110). The planned item set was retained in the measurement model. Competing measurement models that collapsed the downstream constructs fit substantially worse. Merging perceived safety and flow produced chi-square(1304) = 2774.04, CFI = 0.929, TLI = 0.925, and RMSEA = 0.052, with delta chi-square(7) = 1250.61, *p* < 0.001, and delta CFI = −0.060 relative to the eight-factor model. Merging perceived safety, flow, and re-participation intention produced chi-square(1310) = 2952.69, CFI = 0.921, TLI = 0.917, and RMSEA = 0.054, with delta chi-square(13) = 1429.27, *p* < 0.001, and delta CFI = −0.068. These comparisons support measurement-level differentiation of the three downstream constructs (Supplementary Table S6).

Parcel-level invariance screening indicated broadly comparable measurement functioning across activity types and sites. Across activity types, configural, metric, and scalar models yielded CFI values of 0.989, 0.987, and 0.986; RMSEA values of 0.041, 0.043, and 0.043; and SRMR values of 0.031, 0.038, and 0.039. Across sites, the corresponding values were CFI = 0.978, 0.979, and 0.978; RMSEA = 0.058, 0.056, and 0.054; and SRMR = 0.033, 0.042, and 0.043. Changes in CFI did not exceed 0.002 across activities or 0.001 across sites. Several alpha and omega estimates exceeded 0.90, indicating strong consistency but also possible item redundancy.

Common-method diagnostics did not support a single-factor account. The first unrotated factor explained 40.56% of variance across 53 focal items, and a one-factor CFA fit poorly, chi-square(1,325) = 11,570.62, CFI = 0.506, TLI = 0.486, RMSEA = 0.135.

### Structural model

The structural paths were estimated as observed-score path models using standardized construct means (Table 3). T1 risk perception was positively associated with T2 threat appraisal (beta = 0.443, SE = 0.036, 95% CI [0.373, 0.513], *p* < 0.001), and T1 baseline trust was negatively associated with threat appraisal (beta = −0.382, SE = 0.036, 95% CI [−0.452, −0.312], *p* < 0.001). The model explained 60.4% of the variance in threat appraisal.

**Table 3:** Standardized direct structural paths.

Outcome	Predictor	Beta	SE	95% CI	*p*	*R* ^2^
T2 threat appraisal	T1 risk perception	0.443	0.036	[0.373, 0.513]	<0.001	0.604
T2 threat appraisal	T1 baseline trust	−0.382	0.036	[−0.452, −0.312]	<0.001	0.604
T2 threat appraisal	Risk perception x baseline trust	−0.307	0.033	[−0.371, −0.243]	<0.001	0.604
T2 challenge appraisal	T1 risk perception	0.555	0.039	[0.478, 0.633]	<0.001	0.516
T2 challenge appraisal	T1 baseline trust	0.511	0.039	[0.433, 0.588]	<0.001	0.516
T2 challenge appraisal	Risk perception × baseline trust	0.250	0.036	[0.180, 0.321]	<0.001	0.516
T2 perceived safety	T2 threat appraisal	−0.559	0.032	[−0.622, −0.497]	<0.001	0.640
T2 perceived safety	T2 challenge appraisal	0.484	0.031	[0.423, 0.545]	<0.001	0.640
T2 flow	T2 threat appraisal	−0.364	0.046	[−0.453, −0.274]	<0.001	0.577
T2 flow	T2 challenge appraisal	0.496	0.043	[0.413, 0.580]	<0.001	0.577
T2 flow	T2 perceived safety	0.125	0.054	[0.019, 0.230]	0.021	0.577
T3 re-participation intention	T2 perceived safety	0.432	0.036	[0.362, 0.502]	<0.001	0.705
T3 re-participation intention	T2 flow	0.442	0.035	[0.373, 0.511]	<0.001	0.705

Coefficients are standardized observed-score path coefficients adjusted for age, gender, education, income, prior aerial adventure experience, broader outdoor adventure experience, sensation seeking, activity type, and site.

T1 risk perception was also positively associated with T2 challenge appraisal (beta = 0.555, SE = 0.039, 95% CI [0.478, 0.633], *p* < 0.001), and T1 baseline trust was positively associated with challenge appraisal (beta = 0.511, SE = 0.039, 95% CI [0.433, 0.588], *p* < 0.001). The model explained 51.6% of the variance in challenge appraisal.

**Figure 3:**
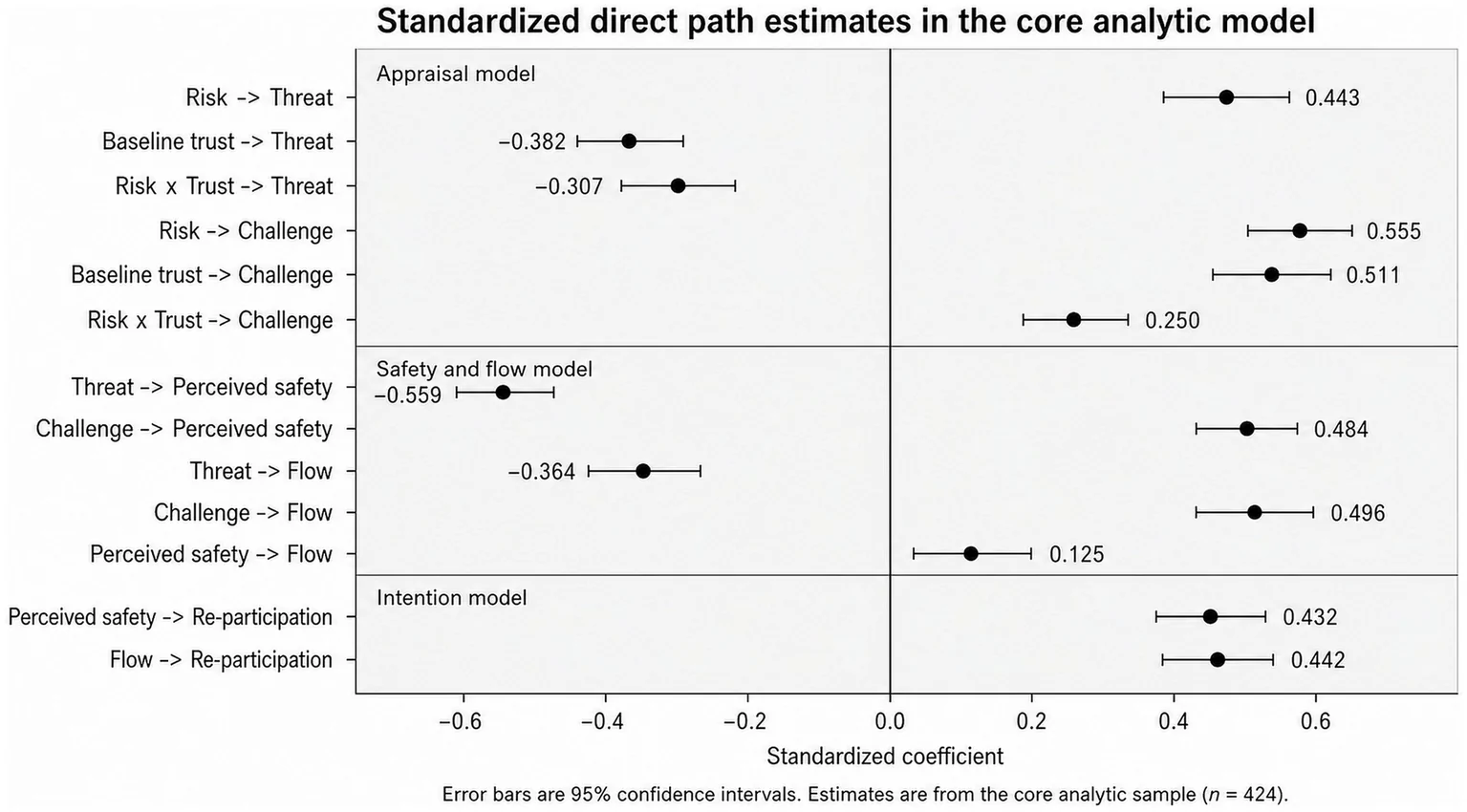
Standardized direct path estimates in the core analytic model. Points represent standardized coefficients and horizontal bars represent 95% confidence intervals. The vertical reference line marks zero. Estimates are based on the retained three-wave sample (*n* = 424) and are adjusted for age, gender, education, income, prior aerial adventure experience, broader outdoor adventure experience, sensation seeking, activity type, and site. Coefficients correspond to the direct paths reported in Table 3.

T2 threat appraisal was negatively associated with perceived safety (beta = −0.559, SE = 0.032, 95% CI [−0.622, −0.497], *p* < 0.001) and flow (beta = −0.364, SE = 0.046, 95% CI [−0.453, −0.274], *p* < 0.001). T2 challenge appraisal was positively associated with perceived safety (beta = 0.484, SE = 0.031, 95% CI [0.423, 0.545], *p* < 0.001) and flow (beta = 0.496, SE = 0.043, 95% CI [0.413, 0.580], *p* < 0.001). T2 perceived safety was positively associated with T2 flow (beta = 0.125, SE = 0.054, 95% CI [0.019, 0.230], *p* = 0.021). Finally, T2 perceived safety (beta = 0.432, SE = 0.036, 95% CI [0.362, 0.502], *p* < 0.001) and T2 flow (beta = 0.442, SE = 0.035, 95% CI [0.373, 0.511], *p* < 0.001) were both positively associated with T3 re-participation intention. The T3 model explained 70.5% of the variance in re-participation intention. Figure 3 visualizes the standardized direct path estimates and their 95% confidence intervals.

### Moderation by baseline trust

**Figure 4:**
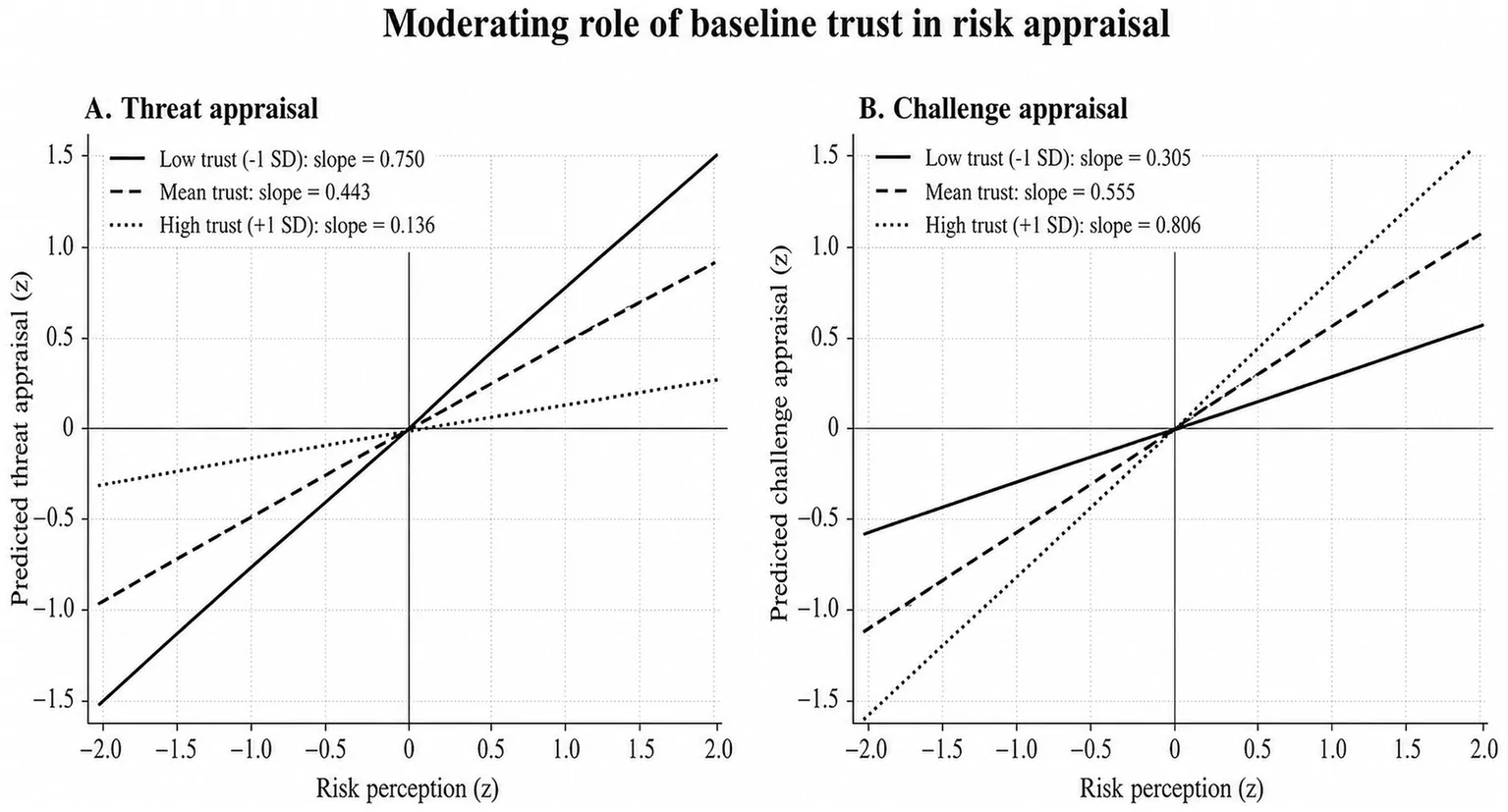
Baseline trust moderates the associations between T1 risk perception and T2 appraisal. Panel **(A)** shows predicted threat appraisal at low (−1 SD), mean, and high (+1 SD) baseline trust. Panel **(B)** shows predicted challenge appraisal at the same baseline-trust levels. The x-axis represents standardized T1 risk perception and the y-axis represents predicted standardized appraisal. Standardized simple slopes indicated that risk perception was most strongly associated with threat under low trust (slope = 0.750, 95% CI [0.654, 0.846], *p* < 0.001), remained positive at mean trust (slope = 0.443, 95% CI [0.372, 0.514], *p* < 0.001), and was weaker but still significant at high trust (slope = 0.136, 95% CI [0.042, 0.230], *p* = 0.005). For challenge appraisal, the association became stronger as baseline trust increased: low trust (slope = 0.305, 95% CI [0.199, 0.411], *p* < 0.001), mean trust (slope = 0.555, 95% CI [0.478, 0.632], *p* < 0.001), and high trust (slope = 0.806, 95% CI [0.702, 0.910], *p* < 0.001).

The risk perception by baseline trust interaction was significant for threat appraisal (beta = −0.307, SE = 0.033, 95% CI [−0.371, −0.243], *p* < 0.001) and challenge appraisal (beta = 0.250, SE = 0.036, 95% CI [0.180, 0.321], *p* < 0.001). The risk-threat slope decreased from low trust (beta = 0.750, SE = 0.049, 95% CI [0.654, 0.846], *p* < 0.001) to mean trust (beta = 0.443, SE = 0.036, 95% CI [0.372, 0.514], *p* < 0.001) and high trust (beta = 0.136, SE = 0.048, 95% CI [0.042, 0.230], *p* = 0.005). Conversely, the risk-challenge slope increased from low trust (beta = 0.305, SE = 0.054, 95% CI [0.199, 0.411], *p* < 0.001) to mean trust (beta = 0.555, SE = 0.039, 95% CI [0.478, 0.632], *p* < 0.001) and high trust (beta = 0.806, SE = 0.053, 95% CI [0.702, 0.910], *p* < 0.001). Figure 4 displays these interactions.

Adjusted marginal means showed the same pattern in scale units. Moving from lower to higher observed risk perception increased threat appraisal by 0.883 points at low trust (*d* = 0.886), 0.553 points at median trust (*d* = 0.555), and 0.279 points at high trust (*d* = 0.280). The corresponding increases in challenge appraisal were 0.624 points (*d* = 0.678), 0.857 points (*d* = 0.931), and 1.052 points (*d* = 1.143). Full adjusted means appear in Supplementary Table S2.

### Indirect and conditional indirect associations

**Figure 5:**
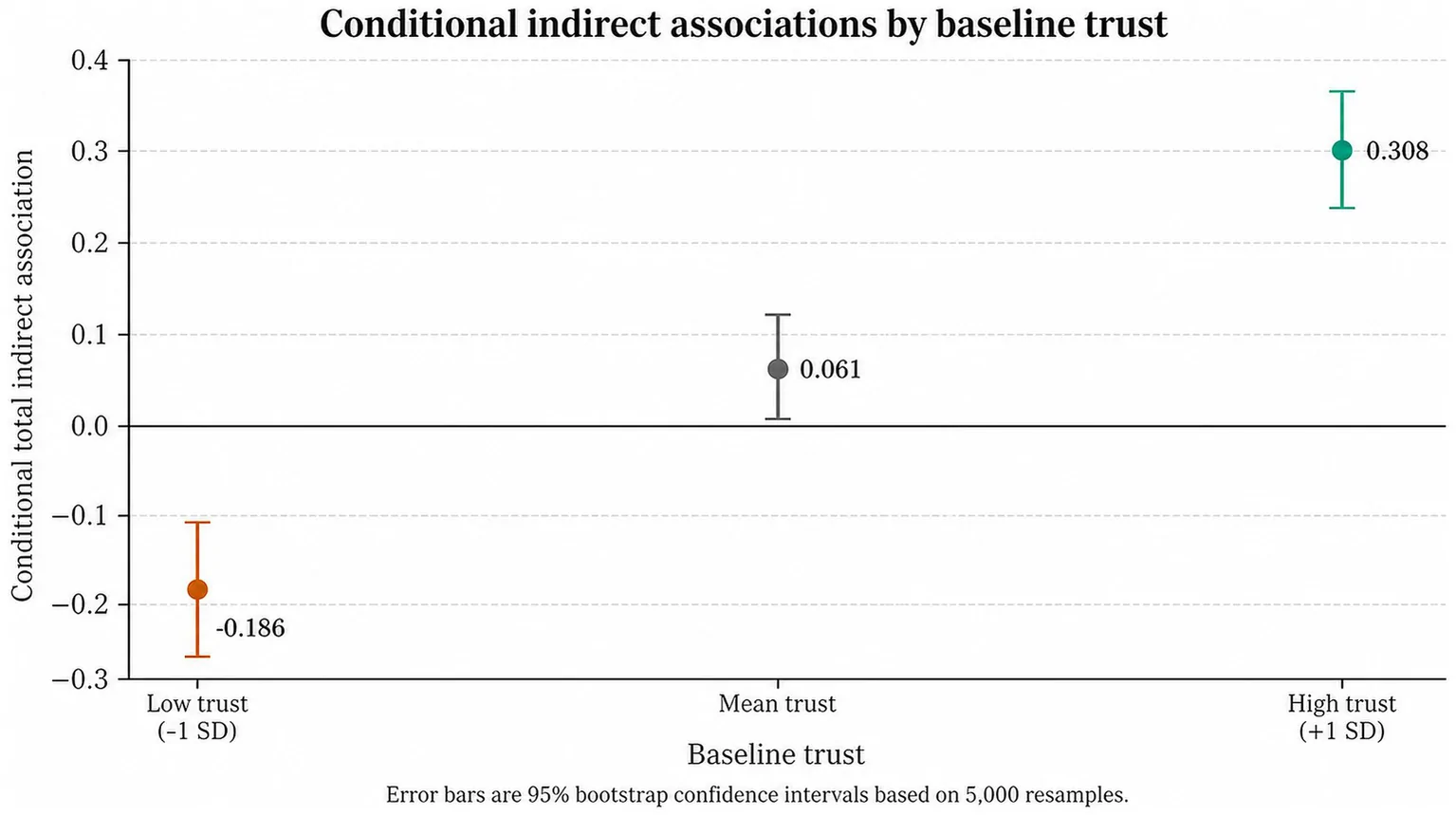
Conditional total indirect associations of *T1* risk perception with *T3* re-participation intention through *T2* appraisal and experience states at low, mean, and high baseline trust. Points represent bootstrap estimates, and vertical error bars represent 95% bootstrap confidence intervals from 5,000 nonparametric resamples. The conditional total indirect association was negative at low trust (estimate = −0.186, 95% CI [−0.256, −0.114]), positive but small at mean trust (estimate = 0.061, 95% CI [0.006, 0.115]), and positive at high trust (estimate = 0.308, 95% CI [0.235, 0.384]). These estimates describe conditional temporal associations in an observational three-wave design.

Bootstrap confidence intervals with 5,000 resamples supported conditional indirect associations from T1 risk perception to T3 re-participation intention through T2 appraisals and experience states. At low baseline trust, the total indirect association was negative (estimate = −0.186, 95% CI [−0.256, −0.114]), reflecting stronger threat-oriented pathways. At mean baseline trust, the total indirect association was small but positive (estimate = 0.061, 95% CI [0.006, 0.115]). At high baseline trust, the total indirect association was more strongly positive (estimate = 0.308, 95% CI [0.235, 0.384]), reflecting stronger challenge-oriented pathways. The main negative threat pathways and positive challenge pathways had bootstrap confidence intervals that did not include zero. Table 4 summarizes conditional associations and robustness analyses, and Figure 5 displays the conditional total indirect associations.

**Table 4:** Conditional associations and robustness analyses.

Analysis	Result
Simple slope: risk perception to threat at low trust	Beta = 0.750, SE = 0.049, 95% CI [0.654, 0.846], *p* < 0.001
Simple slope: risk perception to threat at mean trust	Beta = 0.443, SE = 0.036, 95% CI [0.372, 0.514], *p* < 0.001
Simple slope: risk perception to threat at high trust	Beta = 0.136, SE = 0.048, 95% CI [0.042, 0.230], *p* = 0.005
Simple slope: risk perception to challenge at low trust	Beta = 0.305, SE = 0.054, 95% CI [0.199, 0.411], *p* < 0.001
Simple slope: risk perception to challenge at mean trust	beta = 0.555, SE = 0.039, 95% CI [0.478, 0.632], *p* < 0.001
Simple slope: risk perception to challenge at high trust	Beta = 0.806, SE = 0.053, 95% CI [0.702, 0.910], *p* < 0.001
Total indirect association at low trust	−0.186, 95% bootstrap CI [−0.256, −0.114]
Total indirect association at mean trust	0.061, 95% bootstrap CI [0.006, 0.115]
Total indirect association at high trust	0.308, 95% bootstrap CI [0.235, 0.384]
Common-method diagnostic	First unrotated factor = 40.56%; one-factor CFA: CFI = 0.506, TLI = 0.486, RMSEA = 0.135
Construct-overlap diagnostics	Maximum HTMT = 0.806; merged-factor CFA models fit substantially worse than the eight-factor model; the positive-experience composite matched the simultaneous T3 model at R2 = 0.705, and the safety-flow slope equality test yielded *p* = 0.875 (Supplementary Tables S5, S6)
T3 model plus post-activity trust	Perceived safety beta = 0.400, 95% CI [0.318, 0.483], *p* < 0.001; flow beta = 0.429, 95% CI [0.357, 0.500], *p* < 0.001; post-activity trust beta = 0.058, 95% CI [−0.023, 0.138], *p* = 0.159
Recommendation intention outcome	Perceived safety beta = 0.372, 95% CI [0.292, 0.451], *p* < 0.001; flow beta = 0.498, 95% CI [0.419, 0.576], *p* < 0.001
T1-based inverse-probability weighted T3 model	Perceived safety beta = 0.450, 95% CI [0.382, 0.518], *p* < 0.001; flow beta = 0.423, 95% CI [0.354, 0.492], *p* < 0.001
Post-T2 retention profile and augmented IPW	Retained participants had more favorable T2 states than the 79 participants lost before T3; the T2 block did not improve retention prediction, chi-square(5) = 4.03, *p* = 0.546; augmented-IPW betas = 0.439 for perceived safety and 0.431 for flow, both *p* < 0.001 (Supplementary Table S7)
Activity-type descriptive comparison	Tandem *n* = 202; powered *n* = 133; other comparable *n* = 89; all tandem-versus-powered focal differences were small, absolute *d* ≤ 0.130 (Supplementary Table S8)
HC3 robust SE sensitivity	Focal paths retained direction and significance; weakest focal path was perceived safety to flow, beta = 0.125, robust SE = 0.060, 95% CI [0.007, 0.242], *p* = 0.038
Influential-case sensitivity	Excluding 24 cases with Cook’s *D* > 4/*n*: perceived safety beta = 0.404, 95% CI [0.339, 0.470], *p* < 0.001; flow beta = 0.461, 95% CI [0.398, 0.525], *p* < 0.001
Residual, site, and multicollinearity diagnostics	Breusch-Pagan tests supported HC3 sensitivity checks for selected models; focal site ICC range = 0.000 to 0.030; maximum VIF = 2.73 in the T2 flow model and 1.71 in the T3 re-participation intention model
Leave-one-site sensitivity	T3 perceived safety beta range = 0.396 to 0.481; T3 flow beta range = 0.408 to 0.473; all *p* < 0.001
Leave-one-activity sensitivity	T3 perceived safety beta range = 0.352 to 0.506; T3 flow beta range = 0.375 to 0.545; all *p* < 0.001
Broader field sensitivity sample (*n* = 600)	Included all T3-matched field records, including non-core low-altitude or sightseeing-oriented projects; main directional pattern stable, but perceived safety to flow was weaker and non-significant, beta = 0.073, *p* = 0.104

### Robustness analyses

The main T3 results were stable across alternative outcome and covariate specifications. When T2 post-activity trust was added, perceived safety (beta = 0.400, SE = 0.042, 95% CI [0.318, 0.483], *p* < 0.001) and flow (beta = 0.429, SE = 0.036, 95% CI [0.357, 0.500], *p* < 0.001) remained positive predictors of re-participation intention, whereas post-activity trust was not statistically significant (beta = 0.058, SE = 0.041, 95% CI [−0.023, 0.138], *p* = 0.159). Recommendation intention showed the same pattern: perceived safety (beta = 0.372, SE = 0.040, 95% CI [0.292, 0.451], *p* < 0.001) and flow (beta = 0.498, SE = 0.040, 95% CI [0.419, 0.576], *p* < 0.001) were both positive predictors.

Alternative T3 models quantified predictive overlap among perceived safety, flow, and intention. Perceived-safety-only and flow-only models explained less variance than the simultaneous model (R2 = 0.591 and 0.599 vs. 0.705). An equal-weight positive-experience composite matched the simultaneous model (R2 = 0.705; adjusted R2 = 0.694) with lower AIC and BIC. A Wald contrast did not distinguish the standardized perceived-safety and flow slopes in the simultaneous model, difference = −0.010, SE = 0.064, *p* = 0.875 (Supplementary Tables S5, S6).

The inverse-probability weighting sensitivity analysis also supported the final intention model. Stabilized weights were estimated from observed T1 predictors of retention, including risk perception and baseline trust, and trimmed before estimation. This specification upweighted retained participants who resembled non-retained participants on observed baseline characteristics. In the weighted model, perceived safety (beta = 0.450, SE = 0.035, 95% CI [0.382, 0.518], *p* < 0.001) and flow (beta = 0.423, SE = 0.035, 95% CI [0.354, 0.492], *p* < 0.001) remained positive predictors of T3 re-participation intention. A post-T2 augmented IPW analysis was then estimated among the 503 T2 completers. Adding T2 threat appraisal, challenge appraisal, perceived safety, flow, and post-activity trust did not improve retention prediction beyond the T1 model, but the augmented weights directly incorporated the observed post-activity profile. The weighted T3 associations remained stable for perceived safety (beta = 0.439, SE = 0.035, 95% CI [0.369, 0.509], *p* < 0.001) and flow (beta = 0.431, SE = 0.035, 95% CI [0.362, 0.499], *p* < 0.001). The augmented weights ranged from 0.851 to 2.298 after trimming and yielded an effective sample size of 398.9 (Supplementary Table S7).

Breusch-Pagan tests indicated heteroskedasticity in the challenge appraisal, perceived safety, and flow models (*p* = 0.004–0.023), but not in the threat appraisal or T3 intention models (*p* = 0.434 and 0.170). With HC3 standard errors, the interactions remained significant for threat appraisal (beta = −0.307, robust SE = 0.030, 95% CI [−0.366, −0.247], *p* < 0.001) and challenge appraisal (beta = 0.250, robust SE = 0.039, 95% CI [0.174, 0.327], *p* < 0.001). The perceived safety-flow path also remained positive (beta = 0.125, robust SE = 0.060, 95% CI [0.007, 0.242], *p* = 0.038). Maximum variance inflation factors were 2.73 in the flow model and 1.71 in the intention model; site-level ICCs ranged from 0.000 to 0.030.

Influential-case diagnostics for the final T3 model identified 24 observations above Cook’s *D* > 4/*n* (threshold = 0.0094; maximum Cook’s D = 0.0422). Excluding these observations left the focal T3 results substantively unchanged: perceived safety beta = 0.404, SE = 0.033, 95% CI [0.339, 0.470], *p* < 0.001; flow beta = 0.461, SE = 0.032, 95% CI [0.398, 0.525], *p* < 0.001; *n* = 400. The full retained sample remained the primary analytic specification; the exclusion model was used as an influential-case sensitivity analysis.

Leave-one-site and leave-one-activity checks indicated stability across recruitment contexts. When one site was omitted at a time, perceived safety coefficients ranged from 0.396 to 0.481 and flow coefficients ranged from 0.408 to 0.473, with both predictors remaining significant in all models. When one activity type was omitted at a time, the corresponding ranges were 0.352 to 0.506 for perceived safety and 0.375 to 0.545 for flow. The moderation pattern was similarly stable: the risk perception by baseline trust interaction ranged from −0.320 to −0.294 for threat appraisal and from 0.242 to 0.262 for challenge appraisal in leave-one-site analyses, and from −0.327 to −0.300 and 0.182 to 0.304, respectively, in leave-one-activity analyses. A broader field sensitivity analysis including all T3-matched field records (*n* = 600), including non-core low-altitude or sightseeing-oriented projects, produced the same directional pattern for the main paths, although the perceived safety to flow path was weaker and not statistically significant (beta = 0.073, *p* = 0.104). Descriptive activity profiles in the linked core sample included 202 tandem-paragliding participants, 133 powered-paragliding participants, and 89 participants in other comparable guided aerial adventure activities. Tandem-versus-powered differences were small across risk perception, baseline trust, threat appraisal, challenge appraisal, perceived safety, flow, and re-participation intention (absolute *d* range = 0.002–0.130; all unadjusted Welch *p* values ≥ 0.243). These nonrandomized comparisons provide context rather than evidence of activity equivalence; full values are reported in Supplementary Table S8.

## Discussion

This three-wave field study offers a resource-conditioned account of risk appraisal in guided aerial adventure sport. Pre-activity risk perception was associated with both post-activity threat and challenge, and baseline trust shifted the balance between them: higher trust attenuated the risk-threat association and amplified the risk-challenge association. Threat and challenge appraisals were, in turn, associated in opposite directions with perceived safety and flow, which were both associated with re-participation intention approximately 3 weeks later. The conditional total indirect association changed from negative at low trust to positive at high trust. Measurement models distinguished perceived safety, flow, and intention, whereas the equal-weight composite and slope contrast showed that safety and flow carried substantial shared predictive information. The core contribution is therefore not a claim that risk directly promotes or deters participation, but an account of how trusted social and technical resources shape the meaning assigned to risk.

### Psychological interpretation and theoretical contribution

Risk cues do not carry a fixed psychological valence. Tourism and sport studies commonly treat perceived risk as a barrier to intention or participation (Liang and Xue, 2021; Johann et al., 2022; Li et al., 2023; Nam and Yang, 2025; Teng et al., 2025), yet framing and individual-difference research shows that people interpret uncertainty through psychological distance, prior tendencies, and available resources (Buelow et al., 2022; Xie et al., 2023; Zhang et al., 2023). The present findings align with challenge-threat approaches in which the ratio of demands to perceived resources organizes motivated experience (Meijen et al., 2020; Tomaka and Magoc, 2021; Lin et al., 2026). In guided aerial sport, the same altitude, wind, and bodily arousal can be appraised as vulnerability, challenge, or a mixture of both.

Separating measurement waves adds temporal specificity to this account. Previous studies connect perceived risk with travel and participation decisions (Jiang et al., 2022; Johann et al., 2022; Nam and Yang, 2025; Gu et al., 2026), and aerial-sport research identifies anxiety, confidence, and risk perception as central features of experience (McNeil et al., 2024). Here, anticipatory risk was measured before participation, appraisal and experience immediately afterward, and intention 3 weeks later. This separation clarifies the proposed psychological sequence without converting an observational design into a causal test. The divergent downstream associations of challenge and threat also agree with evidence that they are distinct dimensions rather than a single bipolar state (Rossato et al., 2018) and with research connecting appraisal to wellbeing, anxiety, coping, and performance evaluation (Mansell, 2021; Turner et al., 2021; Chen and Qu, 2023; Jewiss et al., 2023; Karademir et al., 2026).

Baseline trust extends the resource side of appraisal beyond personal confidence. Participants in guided aerial activities depend on a distributed system of pilot expertise, equipment preparation, weather judgment, and operator procedures. Prior tourism studies link trust with risk interpretation and travel decisions (Sarfraz et al., 2022; Ameen et al., 2024). The present moderation pattern indicates that confidence in this social and technical system is associated with how perceived demands are organized psychologically: trust dampens threat-oriented appraisal while strengthening challenge-oriented appraisal. In this context, coping resources are partly interpersonal and institutional, not solely individual.

Perceived safety and flow further connect appraisal with later intention. Flow is an intrinsically rewarding state across sport, adventure recreation, and tourism (Lu et al., 2022; Swann et al., 2022; Jackson et al., 2023), and recent studies link it with repeated participation and psychological recovery (Bayram et al., 2025; Song et al., 2025). Subjective safety was closely coupled with flow in the present data, consistent with research that treats safety judgments as socially constructed and behaviorally relevant (Zou and Yu, 2022; Xie et al., 2025). This association does not imply that objective hazards were absent. It indicates that participants who experienced the activity as professionally supported also reported greater absorption and stronger willingness to return. Because safety and flow were measured together, their directional ordering remains theoretical; their equal predictive slopes and the performance of the positive-experience composite further suggest a shared evaluative component.

### Practical implications

The practical implication is to make both demands and coping resources legible, not to portray aerial adventure as risk-free. Clear explanations of weather decisions, equipment checks, pilot qualifications, emergency procedures, and participant responsibilities can make professional competence observable before flight. Such communication may help participants interpret arousal within a managed challenge while preserving an accurate understanding of risk.

Three operational priorities follow. First, pre-flight briefings should be specific, consistent, and matched by visible safety practices. Second, staff can distinguish normal activation from operational warning signs and clarify what participants can control during launch, flight, and landing. Third, post-activity feedback and follow-up patterns deserve attention: participants lost after T2 reported more threat and less challenge, safety, flow, and post-activity trust than those retained. These recommendations concern communication and experience design alongside objective safety management; they do not substitute for technical standards, operator judgment, or emergency procedures.

### Limitations and future research

The observational design supports temporal ordering but not causal identification. Unmeasured characteristics may influence appraisal, experience, and intention, and all focal constructs were self-reported. The one-factor diagnostics argued against a single general response dimension, but shared mood, acquiescence, social desirability, and positive evaluative responding remain plausible (Podsakoff et al., 2024). Structural models used standardized scale scores and therefore did not correct coefficients or interactions for measurement error. Larger studies should prioritize latent moderated structural equation models or Bayesian estimation, together with direct measurement of positive affect and alternative bi-factor or composite specifications.

Perceived safety and flow were assessed in the same T2 survey. Their association is therefore contemporaneous; reverse ordering, reciprocal reinforcement, and a broader positive-experience process are all compatible with the data. T3 captured intention rather than verified behavior. Linking surveys to booking records or confirmed repeat participation would provide a stronger behavioral outcome where consent, privacy safeguards, and operator cooperation permit.

Attrition also limits generalizability. Retained participants entered the study with lower risk perception and substantially higher trust, and T3 completers reported more favorable T2 experiences than those lost after T2. T1-based and T1 + T2 inverse-probability weighting preserved the focal coefficients, but weighting cannot reconstruct the missing intentions of nonrespondents or address unmeasured reasons for dropout. The sample may consequently overrepresent initially receptive participants and favorably evaluated experiences.

The setting introduces further boundaries. Participants were recruited at guided aerial sites in the Western Sichuan Plateau, and results may differ in lower-altitude settings, solo aerial sport, passive sightseeing, other cultural contexts, or highly experienced populations. Technical weather parameters and exact flight duration were not consistently available; therefore, they were not reported as distributions or modeled. Nonrandom activity selection, site-operator confounding, and heterogeneity within the other-comparable-activity category also preclude claims of activity equivalence. Future multi-site studies should collect standardized weather, duration, waiting-time, crowding, incident, and operator-log data; test item-level invariance; and examine verified return behavior. Carefully designed experimental or quasi-experimental studies could then evaluate risk-communication and trust-building strategies without interfering with operational safety.

## Conclusion

Perceived risk in guided aerial adventure sport did not map uniformly onto avoidance. Its association with later intention depended on baseline trust and on whether participants organized risk as threat or challenge. Low trust aligned perceived risk with a negative indirect pathway, whereas high trust aligned it with challenge, perceived safety, flow, and a positive indirect pathway. By separating anticipatory judgments, post-activity appraisals, and later intention, this study identifies trust in the social and technical system as a psychologically meaningful appraisal resource. Effective communication should neither erase risk nor dramatize it; it should make demands, competence, procedures, and participant resources clear enough for an informed challenge appraisal.

## Data Availability

The original contributions presented in the study are included in the article/Supplementary material, further inquiries can be directed to the corresponding author.
