# Supplementary material for: Baseline trust moderates risk appraisal in guided aerial adventure sports: a three-wave field study
Source: Front Psychol. 2026 Aug 25;17:1868476. doi: 10.3389/fpsyg.2026.1868476 (PMC13550122; doi:10.3389/fpsyg.2026.1868476)
Supplement: Supplementary file 1 [file Data_Sheet_1.PDF]

# Supplementary Material

Title: Baseline Trust Moderates Risk Appraisal in Guided Aerial Adventure Sports: A Three-Wave Field Study

## Supplementary Table S1. Context-adapted item wording and variable mapping

The table reports English wording for context-adapted project items and item-domain mappings for variables used in the manuscript. For the sensation-seeking control measure, the table reports item domains rather than reproducing source-scale wording.

| Construct | Wave | Variable | English reporting wording or item domain | Domain | Response scale |
|---|---|---|---|---|---|
| **Risk perception** | **T1** | **RP1** | **I am concerned that this activity may involve physical injury or bodily discomfort.** | **Physical/health risk** | **1 = strongly disagree to 5 = strongly agree** |
| **Risk perception** | **T1** | **RP2** | **I am concerned that plateau or mountain environmental conditions may increase uncertainty during the activity.** | **Environmental risk** | **1 = strongly disagree to 5 = strongly agree** |
| **Risk perception** | **T1** | **RP3** | **I am concerned that weather or wind conditions may affect the safety of this activity.** | **Weather risk** | **1 = strongly disagree to 5 = strongly agree** |
| **Risk perception** | **T1** | **RP4** | **I am concerned that equipment performance may affect the safety of this activity.** | **Equipment risk** | **1 = strongly disagree to 5 = strongly agree** |
| **Risk perception** | **T1** | **RP5** | **I am concerned that instructor or operator decisions may affect the safety of this activity.** | **Operator risk** | **1 = strongly disagree to 5 = strongly agree** |
| **Risk perception** | **T1** | **RP6** | **I am concerned that emergency response may be difficult if an unexpected situation occurs.** | **Emergency risk** | **1 = strongly disagree to 5 = strongly agree** |
| **Risk perception** | **T1** | **RP7** | **I feel that this activity involves a higher level of uncertainty than ordinary recreational sport.** | **Overall uncertainty** | **1 = strongly disagree to 5 = strongly agree** |
| **Baseline trust** | **T1** | **BT1** | **I believe the instructor has the professional ability needed to guide this activity safely.** | **Instructor competence** | **1 = strongly disagree to 5 = strongly agree** |
| **Baseline trust** | **T1** | **BT2** | **I believe the instructor can make appropriate decisions if conditions change.** | **Instructor competence** | **1 = strongly disagree to 5 = strongly agree** |
| **Baseline trust** | **T1** | **BT3** | **I believe the equipment used in this activity is reliable.** | **Equipment trust** | **1 = strongly disagree to 5 = strongly agree** |
| **Baseline trust** | **T1** | **BT4** | **I believe the equipment has been checked carefully before use.** | **Equipment/procedure trust** | **1 = strongly disagree to 5 = strongly agree** |

| Construct | Wave | Variable | English reporting wording or item domain | Domain | Response scale |
|---|---|---|---|---|---|
| **Baseline trust** | **T1** | **BT5** | **I believe the operator follows clear safety procedures.** | **Procedure trust** | **1 = strongly disagree to 5 = strongly agree** |
| **Baseline trust** | **T1** | **BT6** | **I believe the safety briefing provides information that is useful for participation.** | **Procedure trust** | **1 = strongly disagree to 5 = strongly agree** |
| **Baseline trust** | **T1** | **BT7** | **I believe the operator would prioritize participant safety if a problem occurred.** | **Benevolence/safety priority** | **1 = strongly disagree to 5 = strongly agree** |
| **Baseline trust** | **T1** | **BT8** | **I believe the operator would not hide important risk information from participants.** | **Integrity** | **1 = strongly disagree to 5 = strongly agree** |
| **Baseline trust** | **T1** | **BT9** | **I believe emergency arrangements are available if unexpected situations occur.** | **Emergency trust** | **1 = strongly disagree to 5 = strongly agree** |
| **Baseline trust** | **T1** | **BT10** | **Overall, I trust the people, equipment, and procedures involved in this activity.** | **Global trust** | **1 = strongly disagree to 5 = strongly agree** |
| **Sensation seeking control** | **T1** | **SS1** | **Preference for exciting and intense experiences.** | **Thrill/intensity seeking** | **1 = strongly disagree to 5 = strongly agree** |
| **Sensation seeking control** | **T1** | **SS2** | **Interest in trying unfamiliar experiences.** | **Experience seeking** | **1 = strongly disagree to 5 = strongly agree** |
| **Sensation seeking control** | **T1** | **SS3** | **Enjoyment of activities with uncertainty.** | **Uncertainty preference** | **1 = strongly disagree to 5 = strongly agree** |
| **Sensation seeking control** | **T1** | **SS4** | **Tendency to feel bored with routine activities.** | **Boredom susceptibility** | **1 = strongly disagree to 5 = strongly agree** |
| **Sensation seeking control** | **T1** | **SS5-SS8** | **Remaining brief sensation-seeking domains were retained as control indicators; source wording is not reproduced here.** | **Control-domain indicators** | **1 = strongly disagree to 5 = strongly agree** |
| **Threat appraisal** | **T2** | **TH1** | **During the activity, I felt that the demands of the situation were greater than my ability to cope.** | **Demands greater than resources** | **1 = strongly disagree to 5 = strongly agree** |
| **Threat appraisal** | **T2** | **TH2** | **At some moments, I felt personally threatened by the activity conditions.** | **Threat** | **1 = strongly disagree to 5 = strongly agree** |
| **Threat appraisal** | **T2** | **TH3** | **I felt that unexpected changes would be difficult for me to handle.** | **Low coping** | **1 = strongly disagree to 5 = strongly agree** |
| **Threat appraisal** | **T2** | **TH4** | **I was worried that I might not be able to stay calm during the activity.** | **Emotional threat** | **1 = strongly disagree to 5 = strongly agree** |

| Construct | Wave | Variable | English reporting wording or item domain | Domain | Response scale |
|---|---|---|---|---|---|
| **Threat appraisal** | **T2** | **TH5** | **The activity made me feel vulnerable to things outside my control.** | **Low control** | **1 = strongly disagree to 5 = strongly agree** |
| **Threat appraisal** | **T2** | **TH6** | **I felt that the potential negative consequences of the activity were salient to me.** | **Anticipated harm** | **1 = strongly disagree to 5 = strongly agree** |
| **Challenge appraisal** | **T2** | **CH1** | **During the activity, I felt that the challenge was demanding but manageable.** | **Manageable demand** | **1 = strongly disagree to 5 = strongly agree** |
| **Challenge appraisal** | **T2** | **CH2** | **I felt that the activity gave me an opportunity to test myself.** | **Self-testing** | **1 = strongly disagree to 5 = strongly agree** |
| **Challenge appraisal** | **T2** | **CH3** | **I felt capable of dealing with the demands of the activity.** | **Coping resources** | **1 = strongly disagree to 5 = strongly agree** |
| **Challenge appraisal** | **T2** | **CH4** | **The risk in the activity made the experience more meaningful rather than simply frightening.** | **Meaning** | **1 = strongly disagree to 5 = strongly agree** |
| **Challenge appraisal** | **T2** | **CH5** | **I experienced the activity as an exciting challenge.** | **Challenge excitement** | **1 = strongly disagree to 5 = strongly agree** |
| **Challenge appraisal** | **T2** | **CH6** | **I felt that completing the activity could give me a sense of achievement.** | **Achievement** | **1 = strongly disagree to 5 = strongly agree** |
| **Perceived safety** | **T2** | **PS1** | **I felt protected by the safety arrangements during the activity.** | **Safety arrangement** | **1 = strongly disagree to 5 = strongly agree** |
| **Perceived safety** | **T2** | **PS2** | **I felt that the activity was conducted within a manageable level of risk.** | **Manageable risk** | **1 = strongly disagree to 5 = strongly agree** |
| **Perceived safety** | **T2** | **PS3** | **I felt that the instructor's guidance made the activity safer.** | **Instructor safety** | **1 = strongly disagree to 5 = strongly agree** |
| **Perceived safety** | **T2** | **PS4** | **I felt that the equipment helped keep the activity under control.** | **Equipment safety** | **1 = strongly disagree to 5 = strongly agree** |
| **Perceived safety** | **T2** | **PS5** | **I believed that the operator could respond if an unexpected situation occurred.** | **Emergency confidence** | **1 = strongly disagree to 5 = strongly agree** |
| **Perceived safety** | **T2** | **PS6** | **Overall, I felt safe during the activity.** | **Global perceived safety** | **1 = strongly disagree to 5 = strongly agree** |
| **Flow** | **T2** | **FL1** | **During the activity, my attention was fully focused on what I was doing.** | **Concentration** | **1 = strongly disagree to 5 = strongly agree** |
| **Flow** | **T2** | **FL2** | **I felt absorbed in the activity.** | **Absorption** | **1 = strongly disagree to 5 = strongly agree** |

| Construct | Wave | Variable | English reporting wording or item domain | Domain | Response scale |
|---|---|---|---|---|---|
| Flow | T2 | FL3 | I felt a strong sense of control during the activity. | Sense of control | 1 = strongly disagree to 5 = strongly agree |
| Flow | T2 | FL4 | The challenge of the activity matched my ability to handle it. | Challenge-skill balance | 1 = strongly disagree to 5 = strongly agree |
| Flow | T2 | FL5 | Time seemed to pass differently during the activity. | Altered time perception | 1 = strongly disagree to 5 = strongly agree |
| Flow | T2 | FL6 | The activity itself felt rewarding while I was doing it. | Autotelic experience | 1 = strongly disagree to 5 = strongly agree |
| Flow | T2 | FL7 | I felt physically and mentally engaged in the activity. | Engagement | 1 = strongly disagree to 5 = strongly agree |
| Flow | T2 | FL8 | My actions and the situation seemed to fit together smoothly. | Action-awareness fit | 1 = strongly disagree to 5 = strongly agree |
| Post-activity trust | T2 | AT1 | Based on today's experience, I trust the instructor's professional ability. | Instructor competence | 1 = strongly disagree to 5 = strongly agree |
| Post-activity trust | T2 | AT2 | Based on today's experience, I trust the instructor's judgment during the activity. | Instructor judgment | 1 = strongly disagree to 5 = strongly agree |
| Post-activity trust | T2 | AT3 | Based on today's experience, I trust the equipment used in the activity. | Equipment trust | 1 = strongly disagree to 5 = strongly agree |
| Post-activity trust | T2 | AT4 | Based on today's experience, I trust the operator's safety procedures. | Procedure trust | 1 = strongly disagree to 5 = strongly agree |
| Post-activity trust | T2 | AT5 | Based on today's experience, I believe the operator treated participant safety seriously. | Safety priority | 1 = strongly disagree to 5 = strongly agree |
| Post-activity trust | T2 | AT6 | Overall, today's experience increased my trust in this type of activity. | Global trust | 1 = strongly disagree to 5 = strongly agree |
| Re-participation intention | T3 | RI1 | I intend to participate in the same or a similar aerial adventure sport again in the future. | Intention | 1 = strongly disagree to 5 = strongly agree |
| Re-participation intention | T3 | RI2 | If conditions are appropriate, I would choose this type of activity again. | Choice likelihood | 1 = strongly disagree to 5 = strongly agree |
| Re-participation intention | T3 | RI3 | I am willing to continue participating in this type of aerial adventure sport. | Continuation | 1 = strongly disagree to 5 = strongly agree |
| Re-participation intention | T3 | RI4 | I would consider this activity type as an option for future sport or leisure plans. | Future planning | 1 = strongly disagree to 5 = strongly agree |

| Construct | Wave | Variable | English reporting wording or item domain | Domain | Response scale |
|---|---|---|---|---|---|
| **Recommendation intention** | **T3** | **REC1** | **I would recommend this type of aerial adventure sport to people who are physically suitable for it.** | **Recommendation** | **1 = strongly disagree to 5 = strongly agree** |
| **Recommendation intention** | **T3** | **REC2** | **I would share positive aspects of this experience with friends or family.** | **Positive sharing** | **1 = strongly disagree to 5 = strongly agree** |
| **Recommendation intention** | **T3** | **REC3** | **If someone asked me about this activity, I would describe it as worth considering.** | **Advice** | **1 = strongly disagree to 5 = strongly agree** |
| **Safety premium exploratory items** | **T3** | **WTP1** | **I would be willing to pay somewhat more for visibly stronger safety arrangements in this type of activity.** | **Optional exploratory** | **1 = strongly disagree to 5 = strongly agree** |
| **Safety premium exploratory items** | **T3** | **WTP2** | **Instructor qualification and emergency preparedness would influence my future choice of operator.** | **Optional exploratory** | **1 = strongly disagree to 5 = strongly agree** |

## Supplementary Table S2. Adjusted conditional mediator means and standardized differences

Adjusted means fixed risk perception at its lower and upper quartiles and baseline trust at its lower quartile, median, and upper quartile, averaging over the observed covariate distribution. Standardized differences divide the adjusted mean difference by the observed mediator standard deviation in the core analytic sample.

| Mediator | Baseline trust level | Trust value | Adjusted mean at lower risk (Q1) | Adjusted mean at higher risk (Q3) | Mean difference | Standardized difference (d) |
|---|---|---|---|---|---|---|
| **Threat appraisal** | **Low trust (Q1)** | **3.6** | **2.217** | **3.1** | **0.883** | **0.886** |
| **Threat appraisal** | **Median trust** | **4.2** | **2.123** | **2.677** | **0.553** | **0.555** |
| **Threat appraisal** | **High trust (Q3)** | **4.7** | **2.045** | **2.324** | **0.279** | **0.28** |
| **Challenge appraisal** | **Low trust (Q1)** | **3.6** | **3.378** | **4.002** | **0.624** | **0.678** |
| **Challenge appraisal** | **Median trust** | **4.2** | **3.586** | **4.443** | **0.857** | **0.931** |
| **Challenge appraisal** | **High trust (Q3)** | **4.7** | **3.76** | **4.811** | **1.052** | **1.143** |

## Supplementary Table S3. Parcel-level measurement invariance screening

Parcel-level multi-group CFA screened configural, metric, and scalar invariance across activity type and site for the focal T1 and T2 constructs. These analyses are supplementary diagnostics rather than substitutes for full item-level invariance testing in larger multi-site samples.

| group | model | converged | chisq | df | cfi | tli | rmsea | srmr | delta_cfi | delta_rmsea | delta_srmr |
|---|---|---|---|---|---|---|---|---|---|---|---|
| **activity_group** | **configural** | **TRUE** | **508.149** | **411** | **0.989** | **0.986** | **0.041** | **0.031** | | | |

| group | model | converged | chisq | df | cfi | tli | rmsea | srmr | delta_cfi | delta_rmsea | delta_srmr |
|---|---|---|---|---|---|---|---|---|---|---|---|
| activity_group | metric | TRUE | 549.874 | 437 | 0.987 | 0.985 | 0.043 | 0.038 | -0.002 | 0.002 | 0.007 |
| activity_group | scalar | TRUE | 583.612 | 463 | 0.986 | 0.985 | 0.043 | 0.039 | -0.001 | 0.000 | 0.001 |
| site_id | configural | TRUE | 883.678 | 685 | 0.978 | 0.973 | 0.058 | 0.033 | | | |
| site_id | metric | TRUE | 929.813 | 737 | 0.979 | 0.975 | 0.056 | 0.042 | 0.001 | -0.003 | 0.009 |
| site_id | scalar | TRUE | 983.187 | 789 | 0.978 | 0.977 | 0.054 | 0.043 | -0.000 | -0.002 | 0.001 |

## Supplementary Table S4. Parcel map used for invariance screening

| construct | parcel | items |
|---|---|---|
| rp | rpp1 | rp_1, rp_4, rp_7 |
| rp | rpp2 | rp_2, rp_5 |
| rp | rpp3 | rp_3, rp_6 |
| bt | btp1 | bt_1, bt_4, bt_7, bt_10 |
| bt | btp2 | bt_2, bt_5, bt_8 |
| bt | btp3 | bt_3, bt_6, bt_9 |
| th | thp1 | th_1, th_4 |
| th | thp2 | th_2, th_5 |
| th | thp3 | th_3, th_6 |
| ch | chp1 | ch_1, ch_4 |
| ch | chp2 | ch_2, ch_5 |
| ch | chp3 | ch_3, ch_6 |
| ps | psp1 | ps_1, ps_4 |
| ps | psp2 | ps_2, ps_5 |
| ps | psp3 | ps_3, ps_6 |
| fl | flp1 | fl_1, fl_5 |
| fl | flp2 | fl_2, fl_6 |
| fl | flp3 | fl_3, fl_7 |
| fl | flp4 | fl_4, fl_8 |
| ri | rip1 | ri_1, ri_3 |
| ri | rip2 | ri_2, ri_4 |

## Supplementary Table S5. Competing/alternative-model analysis for the T3 intention model

Competing and alternative models used the same core analytic sample (n = 424), standardized construct means, and covariate set as the primary T3 intention model: age, sex, education, income, prior aerial adventure experience, broader outdoor adventure experience, sensation seeking, activity type, and site. The main competing model treated T2 perceived safety and T2 flow as a single positive-experience composite. AIC and BIC are reported as descriptive diagnostics; these models were used to examine construct overlap rather than to replace the theory-specified simultaneous model.

Panel A. Model-fit diagnostics.

| Model | Focal predictors | R2 | Adjusted R2 | AIC | BIC | Diagnostic reading |
|---|---|---|---|---|---|---|
| **Primary simultaneous model** | **T2 perceived safety; T2 flow** | **0.705** | **0.694** | **718.5** | **787.3** | **Perceived safety and flow were retained together as the theory-specified T3 model.** |
| **Perceived-safety-only model** | **T2 perceived safety** | **0.591** | **0.576** | **855.1** | **919.9** | **Explained less variance than the simultaneous model.** |
| **Flow-only model** | **T2 flow** | **0.599** | **0.584** | **847.0** | **911.8** | **Explained less variance than the simultaneous model.** |
| **Competing positive-experience composite model** | **Mean of T2 perceived safety and T2 flow** | **0.705** | **0.694** | **716.5** | **781.3** | **Matched the simultaneous model's explained variance, indicating substantial shared positive-experience variance.** |
| **Post-activity trust covariate model** | **T2 perceived safety; T2 flow; T2 post-activity trust** | **0.707** | **0.694** | **718.4** | **791.3** | **Tested whether safety and flow remained stable after adjusting for completed-experience trust.** |

Panel B. Focal adjusted coefficients.

| Model | Predictor | beta | SE | 95% CI | p |
|---|---|---|---|---|---|
| **Primary simultaneous model** | **T2 perceived safety** | **0.432** | **0.036** | **[0.362, 0.502]** | **<0.001** |
| **Primary simultaneous model** | **T2 flow** | **0.442** | **0.035** | **[0.373, 0.511]** | **<0.001** |
| **Perceived-safety-only model** | **T2 perceived safety** | **0.705** | **0.033** | **[0.640, 0.771]** | **<0.001** |
| **Flow-only model** | **T2 flow** | **0.703** | **0.033** | **[0.639, 0.767]** | **<0.001** |
| **Competing positive-experience composite model** | **Positive-experience composite** | **0.789** | **0.028** | **[0.734, 0.845]** | **<0.001** |
| **Post-activity trust covariate model** | **T2 perceived safety** | **0.400** | **0.042** | **[0.318, 0.483]** | **<0.001** |
| **Post-activity trust covariate model** | **T2 flow** | **0.429** | **0.036** | **[0.357, 0.500]** | **<0.001** |
| **Post-activity trust covariate model** | **T2 post-activity trust** | **0.058** | **0.041** | **[-0.023, 0.138]** | **0.159** |

Panel C. Equality of the simultaneous perceived-safety and flow slopes.

| Contrast | Difference | SE | t | p |
|---|---|---|---|---|
| **Standardized perceived-safety slope minus standardized flow slope** | **-0.010** | **0.064** | **-0.157** | **0.875** |

Note. R2 = coefficient of determination; AIC = Akaike information criterion; BIC = Bayesian information criterion. At the prediction level, the equal-weight positive-experience composite matched the simultaneous model's explained variance and the two standardized slopes were not distinguishable. These results do not negate the measurement-level differentiation tested in Supplementary Table S6; they show that the two experience states carried substantial shared predictive information for T3 intention.

## Supplementary Table S6. Competing CFA models for downstream construct differentiation

The eight-factor measurement model was compared with models that merged perceived safety with flow and that merged perceived safety, flow, and re-participation intention. All models used the same linked core sample (n = 424) and the same maximum-likelihood covariance-modeling framework as the primary CFA.

| Model | df | chi-square | CFI | TLI | RMSEA | delta chi-square (delta df) | delta CFI | p |
|---|---|---|---|---|---|---|---|---|
| Eight-factor model | 1297 | 1523.42 | 0.989 | 0.988 | 0.020 | - | - | - |
| Seven-factor model: safety and flow merged | 1304 | 2774.04 | 0.929 | 0.925 | 0.052 | 1250.61 (7) | -0.060 | <0.001 |
| Six-factor model: safety, flow, and intention merged | 1310 | 2952.69 | 0.921 | 0.917 | 0.054 | 1429.27 (13) | -0.068 | <0.001 |

Note. Delta statistics compare each merged-factor model with the eight-factor model. Both merged-factor specifications showed substantial fit deterioration, supporting measurement-level differentiation of perceived safety, flow, and re-participation intention. This result is compatible with their strong shared predictive variance in Supplementary Table S5.

## Supplementary Table S7. Post-T2 attrition and augmented inverse-probability weighting

Panel A. T2 experience states among participants retained versus lost at T3.

| T2 construct | Retained at T3, n = 424 M (SD) | Lost before T3, n = 79 M (SD) | Cohen's d, retained minus lost | Welch t (df) | p |
|---|---|---|---|---|---|
| Threat appraisal | 2.57 (1.00) | 3.43 (1.14) | -0.848 | -6.29 (101.2) | <0.001 |
| Challenge appraisal | 3.88 (0.92) | 3.41 (1.07) | 0.505 | 3.72 (100.6) | <0.001 |
| Perceived safety | 4.05 (0.85) | 3.42 (1.06) | 0.718 | 5.06 (97.9) | <0.001 |
| Flow | 3.87 (0.88) | 3.11 (1.15) | 0.815 | 5.57 (96.1) | <0.001 |
| Post-activity trust | 4.07 (0.81) | 3.29 (1.03) | 0.912 | 6.33 (96.9) | <0.001 |

Panel B. Incremental prediction of T3 retention among T2 completers.

| Retention model | n | AUC | Block likelihood-ratio test |
|---|---|---|---|
| T1 demographic, experience, psychological, site, and activity predictors | 503 | 0.873 | Reference |
| T1 predictors plus T2 threat, challenge, perceived safety, flow, and post-activity trust | 503 | 0.874 | chi-square(5) = 4.03, p = 0.546 |

Panel C. Weighted T3 intention models.

| Weight specification | Predictor | beta | SE | 95% CI | p | Trimmed weight range | Effective n |
|---|---|---|---|---|---|---|---|
| T1-based IPW | Perceived safety | 0.450 | 0.035 | [0.382, 0.518] | <0.001 | 0.810-2.685 | 381.1 |
| T1-based IPW | Flow | 0.423 | 0.035 | [0.354, 0.492] | <0.001 | 0.810-2.685 | 381.1 |
| T1+T2 augmented IPW | Perceived safety | 0.439 | 0.035 | [0.369, 0.509] | <0.001 | 0.851-2.298 | 398.9 |
| T1+T2 augmented IPW | Flow | 0.431 | 0.035 | [0.362, 0.499] | <0.001 | 0.851-2.298 | 398.9 |

Note. Panel A reports unadjusted selection diagnostics; d is positive when retained participants scored higher. Panel B compares nested retention models on the same T2-completer frame. The T1-based weights in Panel C were estimated for the 529-member core T1 cohort, whereas the T1+T2 weights were estimated among the 503 T2 completers; both specifications were trimmed at the 1st and 99th percentiles. HC3 sensitivity intervals also excluded zero for perceived safety and flow under the T1-based specification ([0.360, 0.541] and [0.342, 0.505]) and the T1+T2 specification ([0.354, 0.524] and [0.353, 0.508]). The T2 block did not improve retention prediction beyond T1 factors, and the focal T3 associations remained stable under both weight specifications.

## Supplementary Table S8. Descriptive profiles by core activity type

Activity-specific values are reported for the linked core sample. These comparisons are unadjusted and descriptive because activity type was not assigned and remained partly confounded with site and operator context.

| Construct | Tandem paragliding, n = 202 M (SD) | Powered paragliding, n = 133 M (SD) | Other comparable activities, n = 89 M (SD) | Tandem minus powered d | Welch p |
|---|---|---|---|---|---|
| Risk perception | 3.14 (1.07) | 3.14 (1.09) | 3.08 (1.07) | -0.002 | 0.988 |
| Baseline trust | 3.99 (0.85) | 4.02 (0.85) | 4.11 (0.84) | -0.035 | 0.755 |
| Threat appraisal | 2.62 (1.02) | 2.57 (0.99) | 2.47 (0.95) | 0.047 | 0.669 |
| Challenge appraisal | 3.88 (0.91) | 3.90 (0.96) | 3.87 (0.89) | -0.017 | 0.882 |
| Perceived safety | 3.99 (0.87) | 4.10 (0.85) | 4.11 (0.81) | -0.130 | 0.243 |
| Flow | 3.80 (0.89) | 3.90 (0.91) | 3.98 (0.83) | -0.118 | 0.294 |
| Re-participation intention | 3.68 (0.98) | 3.77 (0.92) | 4.05 (0.86) | -0.095 | 0.388 |

Note. No omnibus multiplicity correction was applied because these comparisons were requested as descriptive context rather than treated as confirmatory activity effects. All absolute tandem-versus-powered standardized differences were <= 0.130 and all unadjusted p values were >= 0.243. The other-comparable-activity category is heterogeneous and should not be interpreted as a single standardized activity format.
